# Functional Analysis of the PI3K/AKT/mTOR Pathway Inhibitor, Gedatolisib, Plus Fulvestrant with and Without Palbociclib in Breast Cancer Models

**DOI:** 10.3390/ijms26125844

**Published:** 2025-06-18

**Authors:** Aaron Broege, Stefano Rossetti, Adrish Sen, Ann De La Forest, Laura Davis, Megan Seibel, Arul S. Menon, Sydney Stokke, Allison Macaulay, Jhomary Molden, Lance Laing

**Affiliations:** 1 Celcuity, Inc., 16305 36th Ave N, Suite 100, Minneapolis, MN 55446, USA; 2 Department of Molecular and Cell Biology, University of California, Berkeley, CA 94720, USA; 3 College of Computing, Data Science, and Society, University of California, Berkeley, CA 94720, USA

**Keywords:** PI3K-AKT-mTOR inhibitors, endocrine therapy, CDK4/6 inhibitors, gedatolisib, fulvestrant, palbociclib, breast cancer

## Abstract

Treatment with endocrine therapy (ET) in combination with CDK4/6 inhibitors has improved the outcome of patients with hormone receptor (HR)+/HER2- advanced breast cancer (ABC), but most patients eventually experience disease progression. Since the PI3K-AKT-mTOR (PAM), estrogen receptor (ER), and cyclin-dependent kinase (CDK) pathways are interdependent drivers of HR+/HER2- breast cancer (BC), the simultaneous inhibition of these pathways is expected to enhance anti-tumor control. Here we investigated the molecular and cellular effects of gedatolisib, a multi-target kinase inhibitor of the PAM pathway currently being evaluated in Phase 3 clinical trials, combined with fulvestrant and/or palbociclib in BC cell models. We found that the gedatolisib/fulvestrant/palbociclib triplet inhibited BC cell growth significantly more than the single agents or the palbociclib/fulvestrant doublet, both in vitro and vivo. Specifically, the triplet combination counteracted adaptive responses associated with single drug treatment, such as the reactivation of the CDK-RB-E2F pathway after palbociclib treatment, and inhibited multiple cellular functions, such as cell cycle progression, cell survival, protein synthesis, and glucose metabolism. The triplet combination was effective in treatment-naïve BC cell lines as well as in cell lines adapted to palbociclib and/or fulvestrant, regardless of *PIK3CA/PTEN* genetic alterations. Our findings provide a mechanistic rationale for conducting clinical studies evaluating gedatolisib in combination with CDK4/6 inhibitors and ET in HR+/HER2- ABC.

## 1. Introduction

The PI3K-AKT-mTOR (PAM) signaling pathway plays a central role in regulating multiple cellular functions, including cell survival, cell proliferation, and metabolic homeostasis [1,2,3]. Increased activation of the PAM pathway is frequent in breast cancer (BC) and is often (>30%) associated with alterations of PAM pathway genes, such as activating mutations or amplification of *PIK3CA*, which encodes the PI3Kα catalytic subunit, or loss of function of PTEN, which is the main negative regulator of the PAM pathway [4]. An increased activation of the PAM pathway can contribute to cancer progression by promoting cancer cell survival and proliferation as well as by supporting metabolic, biosynthetic, and energetic adaptations required to sustain cancer cells’ proliferative functions [1,2,3]. The reliance of cancer cells on the PAM pathway has made this pathway an attractive therapeutic target [5,6,7,8,9].

**Figure 1:**
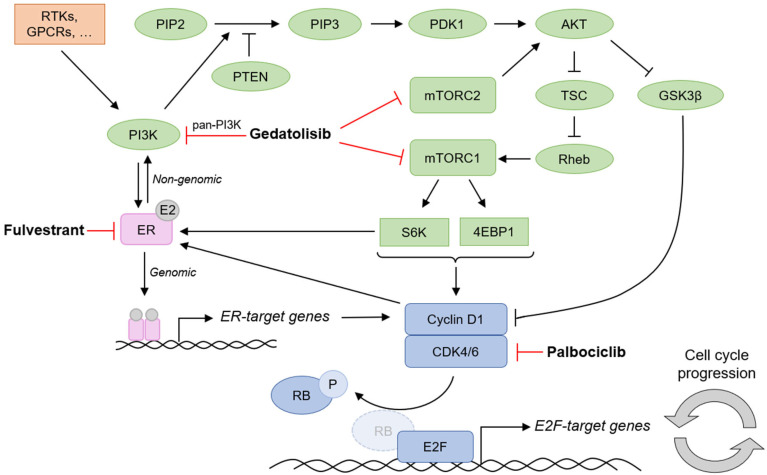
Simplified scheme illustrating the crosstalk between the PI3K-AKT-mTOR (PAM), estrogen receptor (ER), and cyclin-dependent kinase (CDK) 4/6-Retinoblastoma (RB)-E2F pathways and strategies to target these pathways in cancer cells. In response to extracellular stimuli, membrane receptors like receptor tyrosine kinases (RTKs) and G protein-coupled receptors (GPCRs) can activate PI3K, which mediates the conversion of phosphatidylinositol-4, 5-bisphosphate (PIP2) into phosphatidylinositol-3, 4, 5-triphosphate (PIP3). PIP3 accumulation triggers a phosphorylation cascade leading to the activation of multiple effectors, including AKT, which in turn affects several other downstream targets (e.g., mTORC1, GSK3). mTORC2, which also activates AKT, and PTEN, which represses PAM signaling by converting PIP3 to PIP2, are also critical PAM pathway components. The PAM pathway controls various cellular functions, including cell cycle regulation by the cyclin D1-CDK4/6-RB-E2F pathway (e.g., through 4EBP1-mediated translation of cyclin D1). The active cyclin D1-CDK4/6 complex phosphorylates RB, causing its dissociation from the E2F transcription factor; once free from RB, E2F regulates the transcription of genes involved in cell cycle progression. The PAM and CDK4/6 pathways are also interconnected with the estrogen pathway, which plays a critical role in driving cell cycle progression in ER + BC cells. Upon estrogen (E2) binding, ER promotes transcription of ER-target genes involved in multiple cellular functions, including cell cycle. ER can also act non-genomically, e.g., by non-nuclear interaction with PI3K. The crosstalk between these pathways can provide adaptive resistance mechanisms when only one pathway is inhibited. The simultaneous inhibition of the PAM pathway (e.g., with gedatolisib), the CDK4/6 pathway (e.g., with palbociclib), and the ER pathway (e.g., with fulvestrant) is expected to disrupt the cooperation between these pathways and enhance tumor growth inhibition. Scheme based on [10,11].

The increased activation of the PAM pathway is also involved in the development of resistance to drugs used to treat patients with BC, such as endocrine therapy (ET) and CDK4/6 inhibitors, which are the standard of care for patients with hormone receptor-positive (HR+)/HER2-negative (HER2-) advanced breast cancer (ABC). Due to the crosstalk between the PAM, estrogen receptor (ER), and CDK pathways (Figure 1), resistance can arise through adaptive mechanisms when only one of these pathways is inhibited [10,11,12,13,14,15]. For example, resistance to ET has been associated with dysregulation of the CDK4-Rb-E2F transcriptional axis [16]; the CDK4/6 inhibitor palbociclib was shown to decrease the expression of estrogen-regulated genes and reduce sensitivity to ET [17]; resistance to ET and CDK4/6 inhibitors was linked to increased activation of the PAM pathway [18,19]; PI3K inhibition was reported to increase ER transcriptional function [20]; and BC cells resistant to PI3K inhibitors were shown to have sustained CDK4/6 activity [21].

Non-clinical studies have shown that combining PI3K, AKT, or mTOR inhibitors with ET and/or CDK4/6 inhibitors can overcome resistance mechanisms associated with single agent treatments, resulting in increased growth inhibition both in vitro and in vivo [22,23,24,25,26,27]. Evidence that PI3K/mTOR inhibition could reverse resistance to CDK4/6 inhibitors [28] and that ET plus CDK4/6 and PI3K inhibitors was more effective than ET plus CDK4/6 inhibitors in fulvestrant/palbociclib-resistant MCF7 xenograft tumors [24] supports the use of the triplet combination as second-line treatment in patients who progressed after endocrine and CDK4/6 inhibitor therapies. Moreover, non-clinical studies showing that the triplet combination of ET, PAM, and CDK4/6 inhibitors could prevent or delay resistance to single agents in treatment-naïve BC cells [22,26] suggest that this triplet combination would also be effective as first-line therapy [10].

Various PI3K, AKT, and mTOR inhibitors have been tested in combination with ET and/or CDK4/6 inhibitors in clinical trials. Positive outcomes from the pivotal BOLERO-2 [29], SOLAR-1 [30], and CAPItello-291 [31] clinical trials have led to the FDA approval of everolimus (mTORC1 inhibitor), alpelisib (PI3Kα inhibitor), and capivasertib (AKT inhibitor), respectively, when combined with ET for the treatment of patients with HR+/HER2- ABC following progression on or after endocrine therapy. Other clinical trials have also evaluated, or are evaluating, PI3K, AKT, or mTOR inhibitors in combination with endocrine therapy and CDK4/6 inhibitors [10]. While some of these combinations showed acceptable toxicity and preliminary efficacy [32,33], other combinations have shown poorer tolerability [11,34]. The combination of inavolisib, a PI3Kα-selective inhibitor, with palbociclib and fulvestrant was recently approved by the FDA for the treatment of patients with HR+/HER2-, *PIK3CA*-mutated advanced, or metastatic BC [35].

Gedatolisib is a PAM inhibitor targeting all Class I PI3K isoforms, as well as both mTOR complexes, mTORC1 and mTORC2 [36,37]. Gedatolisib has demonstrated in vitro and in vivo efficacy in multiple tumor models, including breast cancer [36,37,38,39,40]. Recent comparative studies have shown that gedatolisib exerted greater anti-proliferative and cytotoxic effects compared to inhibitors targeting single PAM pathway kinases such as alpelisib, capivasertib, and everolimus in multiple cancer cell lines, including BC cell lines [38,39,40]. Compared to these inhibitors, gedatolisib induced a more comprehensive inhibition of the PAM pathway and PAM-controlled functions, including cell proliferation, cell survival, protein synthesis, and glucose metabolism [39,40], thus providing a mechanistic explanation for gedatolisib’s greater efficacy in vitro.

Early-phase clinical studies evaluating gedatolisib in various solid tumors have shown preliminary efficacy and fewer class-associated adverse effects (e.g., hyperglycemia and gastrointestinal and skin toxicities) relative to published data for PI3K or mTOR inhibitors [41,42,43,44,45]. In a Phase 1b clinical trial in patients with HR+/HER2- ABC, the combination of gedatolisib with endocrine therapy (fulvestrant or letrozole) and a CDK inhibitor (palbociclib) showed promising safety and efficacy compared with published data for standard-of-care therapies. Positive results were observed both in patients who had received prior lines of therapy and in treatment-naïve patients [44]. Based on these results, a Phase 3 clinical trial (VIKTORIA-1, NCT05501886) was initiated to evaluate gedatolisib in combination with fulvestrant, with and without palbociclib, as second-line treatment for patients with HR+/HER2- ABC who progressed on or after CDK4/6 and aromatase inhibitor therapy. Another Phase 3 clinical trial (VIKTORIA-2, NCT06757634) was also initiated to evaluate gedatolisib plus a CDK4/6 inhibitor (ribociclib or palbociclib) and fulvestrant as first-line treatment for patients with HR+/HER2- ABC.

In the present study, we assessed the molecular and cellular effects of gedatolisib in combination with fulvestrant, with and without palbociclib, in ER+ breast cancer cell lines with or without *PIK3CA/PTEN* mutations. By employing multiple functional assays, we show that the triplet combination of gedatolisib, fulvestrant, and palbociclib prevented or counteracted early and long-term resistance mechanisms to single drug treatments, resulting in a more durable and effective cell cycle blockade, increased apoptotic cell death, and greater inhibition of metabolic functions compared to treatment with single agents or doublet combinations. As a consequence, the triplet combination also exerted greater anti-proliferative and cytotoxic effects in vitro and induced greater and more durable tumor growth inhibition in vivo compared to treatment with single agents or doublet combinations. The results of this study provide a strong mechanistic rationale for combining gedatolisib, ET, and CDK inhibitors for ABC treatment.

## 2. Results

### 2.1. Concomitant Dysregulation of PAM, ER, and Cell Cycle-Related Pathways in Breast Cancer Patients

**Figure 2:**
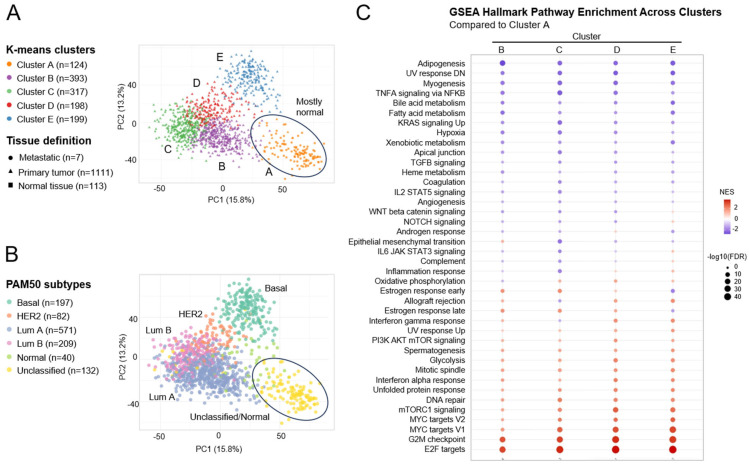
Dimensionality reduction and clustering of 1231 patient tumor samples based on their transcriptomes. (**A**,**B**). k-means clusters overlaid for clinical metadata corresponding to definition and PAM50 subtypes to map clusters to the biological disease progression context. (**C**). Combined dot plot showing pathway enrichment scores in individual clusters following GSEA analysis of Hallmark curated gene sets. NES = normalized enrichment score (NES < 0 indicate negative enrichment relative to cluster A; NES > 0 indicated positive enrichment relative to cluster A).

To assess dynamic changes in molecular pathways during breast cancer progression, we analyzed bulk RNA-seq transcriptional profiles of 1231 breast tumor samples available in the TCGA database. Using unsupervised k-means clustering models on variance-stabilized, batch-corrected patient mRNA profiles, we defined five discrete clusters of tumor transcriptomic states (labeled A through E) (Figure 2A). The analysis of the samples in these clusters by their tissue histology showed that cluster A consisted mostly of normal tissue samples, while clusters B-E overlapped with (primary or metastatic) tumor tissue samples (Figure 2A). These tumor-specific clusters could be further resolved by overlaying PAM50 gene expression signatures [46] to identify the subtype composition for each of the k-means defined clusters. As expected, cluster A was not defined by any of the PAM50 classifiers while luminal A, luminal B, HER2+, and basal PAM50 subtypes could be mapped on to k-means clusters B, C, D, and E, respectively (Figure 2B). Differential gene expression analysis of each tumor-specific cluster (compared to cluster A) revealed several significantly upregulated and downregulated transcripts that characterized clusters B-E (Figure S2). To understand the biological pathways and functions underlying these gene expression differences, gene set enrichment analysis was carried out using the Hallmark and KEGG curated databases (Supplementary Data S1), and subsequently the direction and size of functional changes in different tumor clusters were visualized (Figure 2C). This analysis highlighted that the PAM pathway and cell cycle-related pathways (e.g., E2F-targets, G2/M checkpoint, mitotic spindle) were positively enriched in all clusters relative to normal cells (Figure 2C). Consistent with the typical ER+ status of luminal A and luminal B breast tumors, the ER pathway (early and late estrogen response) was upregulated in cluster B and cluster C (Figure 2C). Several other pathways were identified with positive or negative enrichment during BC progression. For instance, MYC targets, glycolysis, DNA repair, and unfolded protein response were upregulated in all clusters. Other cluster-specific pathway enrichments were also identified, including those associated with EMT (upregulated, cluster B), WNT/beta-catenin/ NOTCH pathway (upregulated, cluster E), and oxidative phosphorylation (downregulated, cluster B) (Figure 2C).

Overall, our bioinformatic analyses highlighted the concomitant dysregulation of PAM, ER, and cell cycle-related pathways in both luminal A and luminal B breast tumors and identified several other functionally related pathways (e.g., glycolysis) whose activity could contribute to BC tumor progression. Co-dysregulation of the PAM, ER, and cell cycle-related pathways in these patient subtypes confirms that the combined targeting of PAM, ER, and CDK pathways is a viable therapeutic strategy in HR + BC.

### 2.2. Growth Inhibitory Effects of Gedatolisib Combined with Fulvestrant and/or Palbociclib in BC Cell Lines

**Figure 3:**
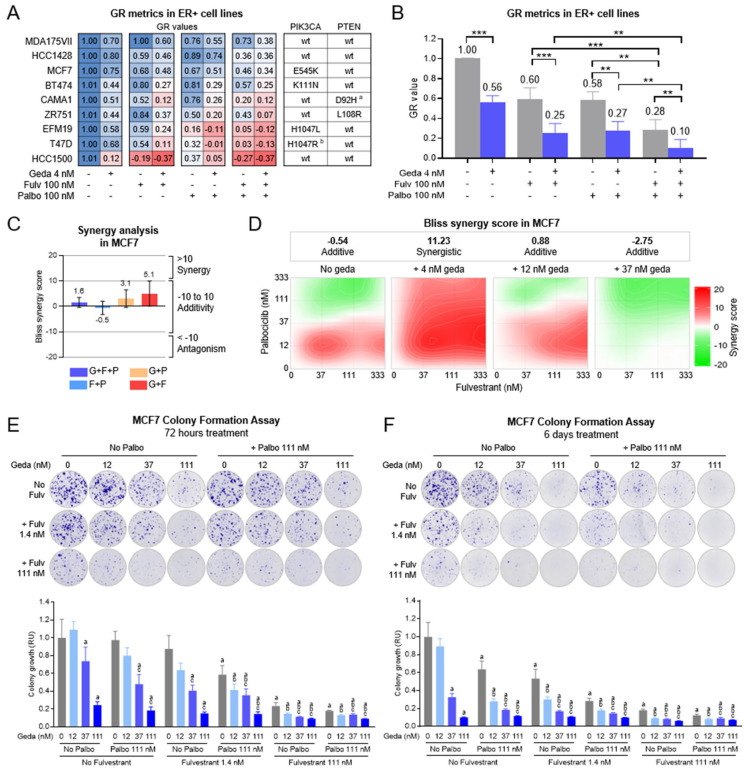
Growth inhibitory effects of gedatolisib plus fulvestrant and/or palbociclib in BC cell lines. (**A**,**B**). BC cell lines treated for 6 days were analyzed for growth rate (GR) inhibition by RTGlo MT cell viability assay. Individual GR values for each cell line are shown in (**A**). GR values between 0 and 1 indicate anti-proliferative effects; GR values = 0 indicate complete cytostatis; GR values < 0 indicate cytotoxic effects, where −1 is complete cell killing. The average GR values for all cell lines +/− SEM are shown in (**B**). ** *p* < 0.01, *** *p* < 0.001 by one-way ANOVA. ^a^ T47D also has *PIK3CA* amplification; ^b^ CAMA1 also has *PTEN* F278Lfs*12 mutation. wt = no driver mutations. (**C**,**D**) SynergyFinder synergy analysis of cell viability in MCF7 cells treated with gedatolisib (G) +/− fulvestrant (F) +/− palbociclib (P) for 6 days. The plot in (**C**) shows overall Bliss synergy scores +/− 95% CI for the various combinations of G, P, and F. Scores > 10 indicate synergy; scores between −10 and 10 indicate additivity; scores < −10 indicate antagonism. The plots in (**D**) show the Bliss synergy score for individual triplet combinations with no geda (i.e., F+P) or geda at 4, 12, and 37 nM. See Supplementary Data S3 for values. (**E**,**F**). Cells treated with gedatolisib, fulvestrant, and/or palbociclib for 72 h (**E**) or 6 days (**F**) were allowed to grow for a total of 2–3 weeks until colonies were visible. Colonies were stained with crystal violet (see micrograph on top) and eluted to quantify colony growth as shown in the graph at the bottom. Data represent mean +/− SEM (*n* = 3). a = *p* < 0.05 vs. DMSO; b = *p* < 0.05 vs. gedatolisib only; c = *p* < 0.05 vs. no gedatolisib within group by two-way ANOVA. See Supplementary Figure S5 for additional statistical analysis and Supplementary Data S4 for values.

Several non-clinical studies have shown that the combination of PAM, CDK4/6, and ER inhibitors exerts greater growth-inhibitory effects than individual drugs in ER + BC cell lines [22,24,25,26,27]. To evaluate the benefit of combining gedatolisib with an ER inhibitor (fulvestrant) and/or a CDK4/6 inhibitor (palbociclib), we initially screened a panel of nine ER + BC cell lines with various PAM pathway mutational status. The cell lines were treated with 4 nM gedatolisib, 100 nM palbociclib, and/or 100 nM fulvestrant for 6 days and analyzed by growth rate (GR) metrics. These concentrations were chosen because, on average, they induced only partial growth rate inhibition (40–44%, Figure 3A,B), which allowed the assessment of potential additive effects of the various drug combinations. On average, the gedatolisib/fulvestrant, gedatolisib/palbociclib, or palbociclib/fulvestrant doublets induced significantly greater (72–75%, *p* < 0.01) growth rate inhibition relative to the single drugs (Figure 3B). The gedatolisib/fulvestrant/palbociclib triplet further increased growth rate inhibition to 90% (*p* < 0.01) (Figure 3B). Furthermore, the combination of gedatolisib with fulvestrant and palbociclib was more effective at inhibiting cell growth than the combination of alpelisib, capivasertib, or everolimus with palbociclib and fulvestrant (Figure S3). Notably, by using a machine learning approach to integrate the panel of experimentally tested cell lines into our clinical BC transcriptomic model, we found that all nine cell lines closely overlapped with k-means clusters B and C (corresponding to clinical luminal A and B PAM50 subtypes, respectively) (Figure S4). This analysis suggests that the cell lines used in our studies are representative of ER + BC tumor subtypes.

To determine if the triplet combination had synergistic effects, we focused on the MCF7 cell line, a *PIK3CA* mutant line used in previous studies to test the effects of similar drug combinations [22,24,25,26,27]. Cells were treated with increasing concentrations of gedatolisib, fulvestrant, and/or palbociclib, and analyzed for cell viability to determine the Bliss synergy score (scores between −10 and 10 = additivity; scores > 10 = synergy). As shown in Figure 3C, the doublet and triplet combinations had an overall additive effect (scores between −0.5 and 5.1). Triplet combination synergy was detected when gedatolisib was used at 4.1 nM (score 11.23); at lower or higher gedatolisib concentration, the triplet combination had additive effects (scores between −2.75 and 0.88) (Figure 3D).

We next used a colony formation assay to test whether the gedatolisib/fulvestrant/palbociclib triplet could induce long-term MCF7 growth inhibition. Sparsely seeded single cells were treated for 72 h or 6 days, washed, and monitored for a total of 2–3 weeks until discrete colonies expanded in the vehicle-treated wells. As shown in Figure 3E, a 72 h treatment with 12–111 nM gedatolisib, 1.4–111 nM fulvestrant, or 111 nM palbociclib as single agents was insufficient to completely abrogate colony growth. Increasing the treatment time to 6 days (with a drug refresh after 72 h) improved the growth-inhibitory effect of all drugs but did not completely abrogate colony growth (Figure 3F). The combination of gedatolisib plus fulvestrant and/or palbociclib inhibited colony formation more effectively than the single drugs. For instance, a 6-day treatment with 12 nM gedatolisib reduced colony formation by 11% versus 72%, 70%, and 82% for gedatolisib in combination with 111 nM palbociclib, 1.4. nM fulvestrant, or both, respectively (Figure 3F).

These studies indicated that the gedatolisib/fulvestrant/palbociclib triplet exerted greater growth inhibitory effects than the single agents or any of the doublet combinations. Moreover, these effects persisted over time and helped prevent the growth of potentially resistant colonies after treatment with the single agents. To investigate the mechanism at the basis of these results, we performed a series of functional assays in BC cells lines with or without *PIK3CA/PTEN* mutations, including MCF7 (*PIK3CA* mutant) and HCC1428 (*PIK3CA/PTEN* wild type).

### 2.3. Effects of the Gedatolisib/Fulvestrant/Palbociclib Triplet on PAM and CDK Pathway Activity

Non-clinical studies have demonstrated that the acquisition of resistance to ET and CDK4/6 inhibitors can involve early adaptations that allow cancer cells to escape growth inhibition [22,47,48,49]. To evaluate whether gedatolisib can counteract early adaptive mechanisms that could contribute to CDK4/6 inhibitor and ET resistance, MCF7 (ER+, *PIK3CA* mut) and HCC1428 (ER+, *PIK3CA/PTEN* wt) cells were treated with various combinations of gedatolisib, fulvestrant, and palbociclib for 24 or 72 h and analyzed by multiple metrics. We initially tested the drug effects on PAM and CDK pathway activities since increased activation of these pathways is a known resistance mechanism to ET and CDK4/6 inhibitors [12,13].

**Figure 4:**
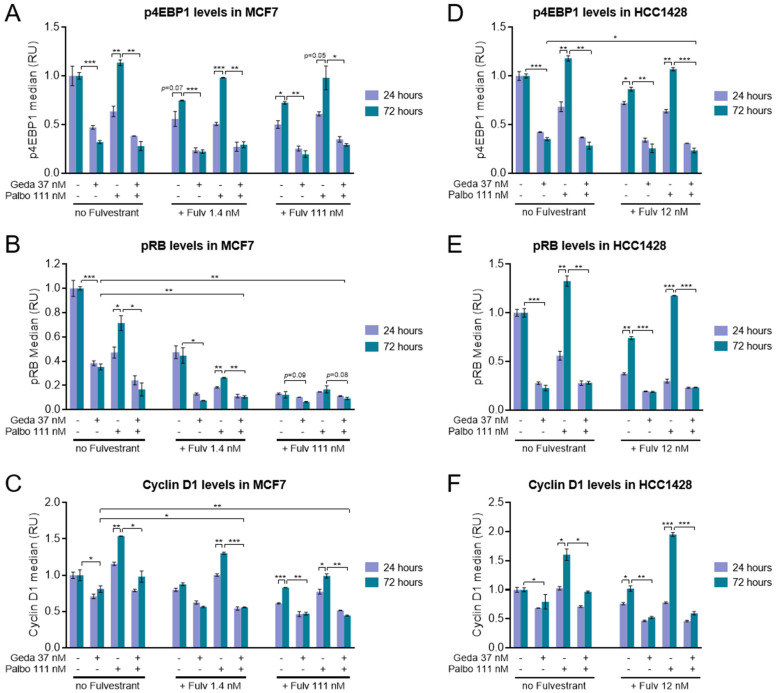
Effects of the gedatolisib/fulvestrant/palbociclib triplet combination on PAM and CDK pathway activity. (**A**–**C**). Flow cytometric analysis of PAM pathway activity (assessed by p4EBP1 staining) (**A**), CDK pathway activity (assessed by pRB staining) (**B**), and cyclin D1 (**C**) in MCF7 cells treated with the indicated concentrations of gedatolisib, fulvestrant, and/or palbociclib for 24–72 h. Data were calculated from the median fluorescence intensity in live cells and are relative to DMSO-treated cells (set as 1). (**D**,**E**). Flow cytometric analysis of p4EBP1 (**D**), pRB (**E**), and cyclin D1 (**F**) in HCC1428 cells as described for (**A**–**C**). Data represent mean +/− SD (*n* = 2). * *p* < 0.05, ** *p* < 0.01, *** *p* < 0.001 by unpaired, two-sided *t*-test. See Supplementary Data S5 for values.

The PAM pathway activity was assessed by flow cytometry analysis of p4EBP1, an effector downstream of PI3K/AKT/mTORC1 previously shown to correlate with gedatolisib growth-inhibitory effects [39]. As expected, gedatolisib (37 nM) reduced p4EBP1 levels by ~50–70% both at 24 and 72 h in MCF7 cells. In contrast, p4EBP1 levels were reduced by 111 nM palbociclib and/or 111 nM fulvestrant at 24 h but significantly increased from 24 h to 72 h. This 72 h recovery was effectively inhibited by the combination with gedatolisib (Figure 4A).

Parallel flow cytometry analysis of pRB levels further demonstrated that gedatolisib and fulvestrant were also effective at suppressing the CDK activity that recovered after 72 h palbociclib treatment. At 1.4 nM fulvestrant and 111 nM palbociclib, the gedatolisib/palbociclib and gedatolisib/fulvestrant doublets and the gedatolisib/fulvestrant/palbociclib triplet inhibited pRB significantly more than the single agents (>80% inhibition versus ~30–65%, *p* < 0.05) (Figure 4B). Fulvestrant applied at a higher concentration (111 nM) inhibited pRB very effectively as a single agent, and adding gedatolisib had less significant effects (*p* < 0.1) (Figure 4B). Consistent with these observations, the combination of gedatolisib with fulvestrant and/or palbociclib was also effective at reducing the increase in cyclin D1 (Figure 4C) and E2F-target gene transcription (*E2F1* and *CDC6*) (Figure S6A) occurring from 24 to 72 h treatment with palbociclib.

Similar results were obtained in HCC1428 cells, where the 72 h recovery in PAM pathway activity (Figure 4D), pRB (Figure 4E), cyclin D1 (Figure 4F), and E2F-target genes (Figure S6B) in response to 111 nM palbociclib and/or 12 nM fulvestrant was significantly suppressed by co-treatment with 37 nM gedatolisib. Of note, the 72 h reactivation of the CDK-RB-E2F pathway after treatment with the fulvestrant/palbociclib doublet was much more pronounced in HCC1428 than in MCF7 cells and was completely abrogated by the co-treatment with gedatolisib.

These results indicated that the gedatolisib/fulvestrant/palbociclib triplet counteracted early adaptations related to the restoration of PAM and CDK pathway activity during treatment with ER and/or CDK4/6 inhibitors. We next performed a series of experiments aimed at testing the effects of the triplet combination on additional cellular functions controlled by PAM/ER/CDK pathways and involved in cancer initiation and progression, including cell cycle, cell survival, protein synthesis, and glucose metabolism.

### 2.4. Effects of the Drug Triplet on Cell Cycling

The restoration of the CDK-RB-E2F pathway activity after 72 h treatment with fulvestrant and/or palbociclib suggested that these early adaptive mechanisms may lead to the restoration of cell cycling, which could be prevented by co-treatment with gedatolisib. Indeed, it has been shown that CDK4/6 inhibitors efficiently block cell cycling upon first exposure, but early adaptations and long-term resistance mechanisms can restore cell cycle progression. PAM pathway inhibition can prevent or counteract some of these resistance mechanisms and induce a more efficient cell cycle blockade [22,25,28].

**Figure 5:**
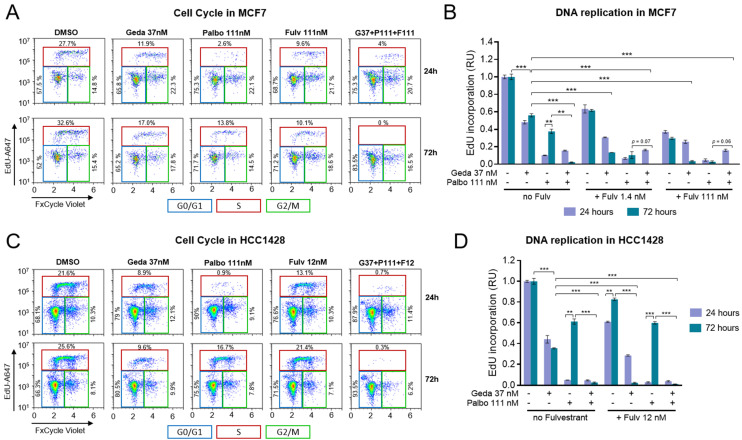
Effects of the gedatolisib/fulvestrant/palbociclib triplet combination on MCF7 and HCC1428 cell proliferation. (**A**,**B**). MCF7 cells were treated with the indicated concentrations of gedatolisib, fulvestrant, and/or palbociclib for 24–72 h and incubated with EdU for the last 2 h of treatment. The cell cycle phases were identified by flow cytometry analysis of EdU incorporation (EdU-A647 staining) and DNA content (FxCycle Violet staining) in Zombie-negative live cells as shown for select drug treatments in (**A**). Quantification of DNA replication (EdU-incorporation) is shown in (**B**). Data represent mean +/− SD (*n* = 2). (**C**,**D**). Flow cytometry analysis of cell cycle (**C**) and DNA replication (**D**) in HCC1428 treated with the indicated drugs for 24–72 h. Data represent mean +/− SD (*n* = 2). ** *p* < 0.01, *** *p* < 0.001 by unpaired, two-sided *t*-test. See Supplementary Data S6 for values.

To assess the effects of the gedatolisib/fulvestrant/palbociclib triplet on cell cycle progression and DNA replication, MCF7 and HCC1428 cells were treated with various combinations of gedatolisib, fulvestrant, and/or palbociclib for 24 or 72 h and analyzed for DNA replication and cell cycle progression by EdU incorporation assay and FxCycle DNA staining. Representative flow cytometry plots identifying the cell cycle phases in response to drug treatments are shown for MCF7 in Figure 5A, while the analysis of DNA replication by EdU incorporation is shown in Figure 5B. In MCF7 cells, 37 nM gedatolisib and 1.4–111 nM fulvestrant reduced relative EdU incorporation by ~50% and ~40–70%, respectively, with little difference between 24 h and 72 h treatments (Figure 5B). As expected, EdU incorporation was substantially reduced by 111 nM palbociclib at 24 h (~90% inhibition) but was partially restored after 72 h (~60% inhibition) (Figure 5B). Resumption of cell cycling after 72 h treatment with palbociclib was effectively counteracted by adding fulvestrant, gedatolisib, or fulvestrant plus gedatolisib, with the triplet combination inducing complete cell cycle blockade at 72 h (Figure 5B). Similar results were observed in HCC1428 cells, where the 72 h cell cycle recovery was observed after treatment with 111 nM palbociclib, 12 nM fulvestrant, or both drugs combined, and was almost completely countered by addition of 37 nM gedatolisib (Figure 5C,D).

These results showed that the gedatolisib/fulvestrant/palbociclib triplet exerted a more efficient and durable cell cycle blockade than the single drugs, which was consistent with the greater inhibition of CDK pathway activity observed previously (see Figure 4).

### 2.5. Effects of the Drug Triplet on Cell Death and Apoptosis

**Figure 6:**
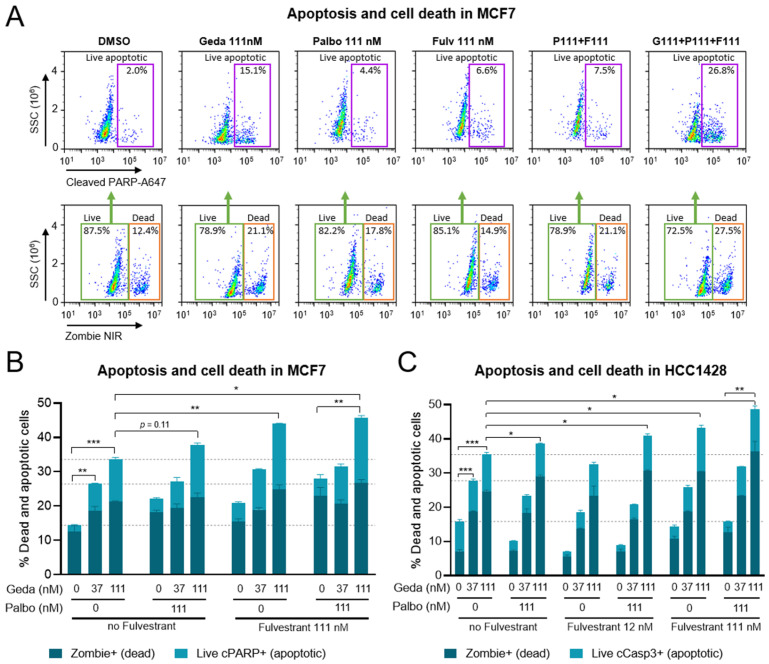
Effects of the gedatolisib/fulvestrant/palbociclib triplet combination on MCF7 and HCC1428 cell survival. (**A**,**B**). MCF7 cells were treated with the indicated concentrations of gedatolisib, fulvestrant, and/or palbociclib for 72 h and analyzed by flow cytometry for cell death (Zombie-positive cells) and apoptosis in live cells (cleaved PARP staining in Zombie-negative cells) as shown in (**A**) for select conditions. Quantification of dead cells (% parent) and live apoptotic cells (% grandparents) in response to the various drug treatments is shown in (**B**). Data represent mean +/− SD (*n* = 2). (**C**). Flow cytometry analysis of cell death (assessed by Zombie staining) and apoptosis (assessed by cleaved Caspase 3 staining in live cells) in HCC1428 treated with the indicated drugs for 72 h. Data represent mean +/− SD (*n* = 2). * *p* < 0.05, ** *p* < 0.01, *** *p* < 0.001 by unpaired, two-sided *t*-test. See Supplementary Data S7 for values.

Concomitant inhibition of the PAM, ER, and CDK pathways has been shown to induce apoptotic cell death in BC cells [22,24,50]. To test the pro-apoptotic, cytotoxic effects of the gedatolisib/fulvestrant/palbociclib triplet, cells were treated with gedatolisib at 37–111 nM in combination with 111 nM fulvestrant and/or 111 nM palbociclib for 72 h and analyzed for cell death and apoptosis by flow cytometry. Cells were first gated by Zombie staining to discriminate live and dead cells; then, the live cells were further gated based on cleaved PARP levels to identify live apoptotic cells (Figure 6A). In live MCF7 cells, gedatolisib increased cleaved PARP levels in a dose- and time-dependent manner from approximately 2% (DMSO control) to 15% (111 nM gedatolisib). Palbociclib (111 nM), fulvestrant (111 nM), and the fulvestrant/palbociclib doublet induced a more modest increase in cleaved PARP (<8%), while the triplet gedatolisib/fulvestrant/palbociclib induced significantly more apoptosis than gedatolisib alone or the fulvestrant/palbociclib doublet (26.8% versus 15.1% and 7.5%, respectively, with 111 nM gedatolisib) (Figure 6A). Analysis of overt cell death by Zombie staining further confirmed that the triplet combination induced more cell death relative to gedatolisib alone (approximately 27.5% versus 21% with 111 nM gedatolisib) (Figure 6A). The total percentage of dead + live apoptotic cells was 46% after treatment with the triplet, compared to 28% and 34% after treatment with the fulvestrant/palbociclib doublet or gedatolisib (111 nM) alone, respectively (Figure 6B). Similar results were obtained in HCC1428 cells (Figure 6C).

These results confirmed that gedatolisib exerted cytotoxic effects as a single agent, and that its combination with fulvestrant (with or without palbociclib) significantly enhanced apoptotic cell death.

### 2.6. Effects of the Drug Triplet on Protein Synthesis

Another important functional process promoted by PAM pathway activation in cancer cells is mTORC1-regulated protein synthesis, which supports cell proliferation and adaptation to stress [51,52]. We previously showed that PAM pathway inhibition by gedatolisib significantly reduced protein synthesis in BC cells [39]. Since estrogen signaling can also control translation (e.g., by regulating the transcription of components of the translation machinery or through the interplay with interconnected pathways) [53], we tested whether fulvestrant, with or without palbociclib, could increase inhibition of protein synthesis when combined with gedatolisib.

**Figure 7:**
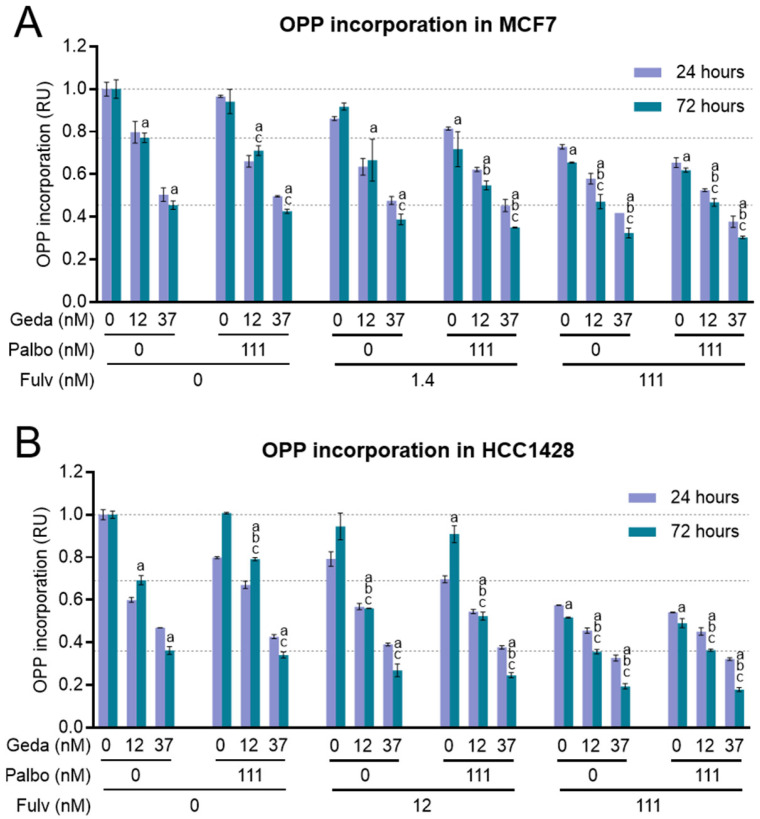
Effects of the gedatolisib/fulvestrant/palbociclib triplet on protein synthesis. (**A**,**B**). MCF7 (**A**) and HCC1428 (**B**) cells were treated with the indicated concentrations of gedatolisib, palbociclib, and/or fulvestrant for 24 or 72 h and incubated with OPP for the last 30 min of treatment. OPP incorporation into newly synthesized proteins was quantified by flow cytometry in live cells, which were gated based on Zombie staining. Data represent mean +/− SD (*n* = 2). a = *p* < 0.05 vs. DMSO; b = *p* < 0.05 vs. gedatolisib only; c = *p* < 0.05 vs. no gedatolisib within group by unpaired, two-sided *t*-test. See Supplementary Data S8 for values.

Protein synthesis was assessed in MCF7 and HCC1428 cells treated with gedatolisib (12–37 nM) in combination with palbociclib (111 nM) and/or fulvestrant (1.4–111 nM for MCF7, 12–111 nM for HCC1428) for 24 or 72 h by using the OPP incorporation assay. The results are shown in Figure 7. As expected, gedatolisib induced a dose-dependent decrease in OPP incorporation in both cell lines, and there was no significant difference between 24 and 72 h. Palbociclib did not affect OPP incorporation in MCF7 and induced a transient 20% reduction at 24 h in HCC1428. Fulvestrant induced a significant and durable decrease in OPP incorporation only at 111 nM. The combination of fulvestrant and gedatolisib significantly increased the inhibition of OPP incorporation relative to single agents (e.g., 23% with 12 nM gedatolisib versus 53% with gedatolisib + 111 nM fulvestrant at 72 h in MCF7 cells; 31% with 12 nM gedatolisib versus 64% with gedatolisib + 111 nM fulvestrant at 72 h in HCC1428 cells). The addition of palbociclib to gedatolisib or to the gedatolisib/fulvestrant doublet had little or no effect.

### 2.7. Effects of the Drug Triplet on Glucose Metabolism

The involvement of the PAM pathway in the regulation of glucose metabolism and glycolysis is well established [2]. In addition, estrogen signaling can also affect glucose uptake and metabolism by controlling the expression of metabolic enzymes or by interacting with other pathways [54]. Our bioinformatics analysis consistently found positive transcript enrichment of the glycolytic pathway, along with the PAM and ER pathways, in luminal A/B breast cancer samples versus normal samples (see Figure 2C).

**Figure 8:**
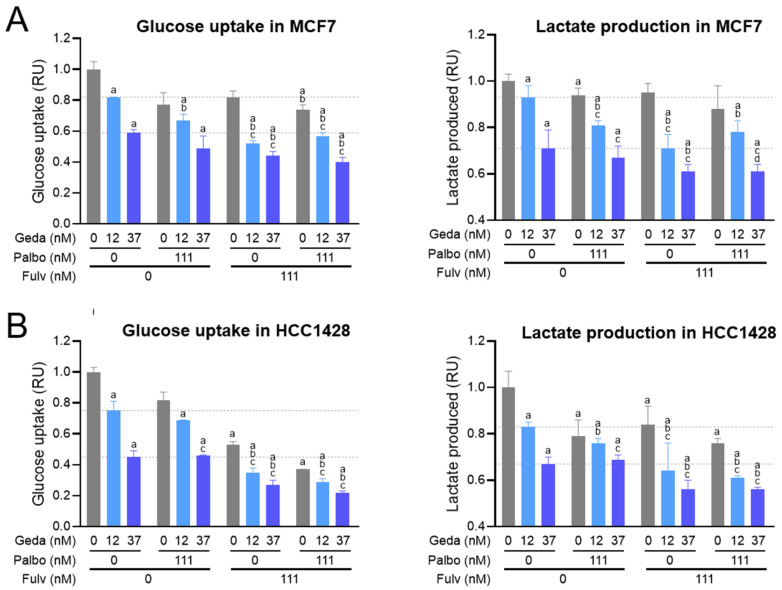
Effects of the gedatolisib/fulvestrant/palbociclib triplet combination on glucose metabolism. (**A**,**B**). MCF7 (**A**) and HCC1428 (**B**) cells were treated with the indicated concentrations of gedatolisib, palbociclib, and/or fulvestrant for 24 h and analyzed for glucose uptake and lactate production. Glucose uptake was quantified by Glucose uptake Glo assay, while lactate production was calculated from the lactate levels measured in the medium with the Biosen R-line instrument before and after treatment. The data shown are normalized to cell number (assessed by BCA analysis) and are relative to DMSO-treated cells (set as 1). Data represent mean +/− SD (*n* = 2). a = *p* < 0.05 vs. DMSO; b = *p* < 0.05 vs. gedatolisib only; c = *p* < 0.05 vs. no gedatolisib within group by unpaired, d = *p* 0.052 versus gedatolisib only; unpaired two-sided *t*-test. See Supplementary Data S9 for values.

We previously demonstrated that gedatolisib inhibits glucose consumption and glycolysis in BC cells [39]. To test the effect of gedatolisib plus fulvestrant, with or without palbociclib, on glucose metabolism, MCF7 and HCC1428 cells were treated with the three drugs in various combinations for 24 h and tested for glucose uptake and lactate production, the end product of glycolysis. As shown in Figure 8, gedatolisib significantly decreased glucose uptake and lactate production in both MCF7 and HCC1428. In both cell lines, the addition of fulvestrant, with or without palbociclib, significantly increased gedatolisib inhibition of glucose uptake and lactate production. For instance, in MCF7, glucose uptake was inhibited by 18% with 12 nM gedatolisib or 111 nM fulvestrant alone compared to 48% inhibition with the two dugs combined (Figure 8A); in HCC1428, glucose uptake was inhibited by 25%, 47%, and 65% with 12 nM gedatolisib, 111 nM fulvestrant, and the two drugs combined, respectively (Figure 8B). In both cell lines, the addition of palbociclib to gedatolisib or to the gedatolisib/fulvestrant doublet had only modest effects.

Overall, the functional analyses performed on BC cells showed that the gedatolisib/fulvestrant/palbociclib triplet increased and/or prolonged the inhibitory effects of the single drugs on multiple pathways and cell functions critical for tumor initiation and progression, such as cell cycle, cell survival, protein synthesis, and glucose metabolism.

### 2.8. Efficacy of the Drug Triplet in a Mouse Model

**Figure 9:**
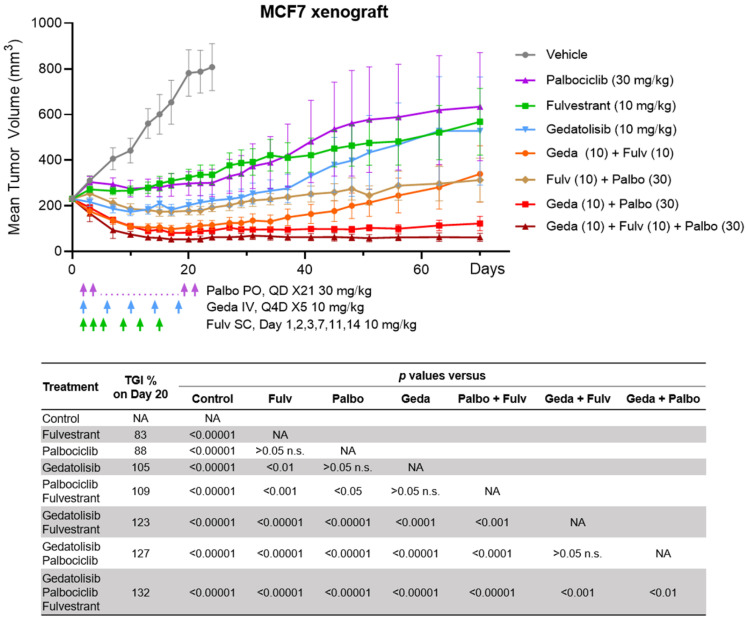
In vivo efficacy of the gedatolisib/fulvestrant/palbociclib triplet. MCF7 cells were injected orthotopically into the mammary fat pad of SCID mice. Mice were treated with vehicle, fulvestrant, palbociclib, gedatolisib, or combinations thereof for 21 days, and tumors were monitored for up to 70 days. The chart on top shows mean tumor volume +/− standard error for each group (*n* = 10). The table at the bottom reports % tumor growth inhibition (TGI) and *p*-values for the individual arm (ANCOVA test). IV = Intravenous; n. s. = not significant; PO = Oral; QDx21 = once daily for 21 days; Q4Dx5 = Once every 4 days for 5 total doses; SC = Subcutaneous. See Supplementary Data S10 for individual tumor volumes.

The growth inhibitory effects of gedatolisib in combination with fulvestrant and gedatolisib observed in vitro were confirmed in vivo using the MCF7 orthotopic BC xenograft mouse model. When the tumor size reached 230 mm^3^, animals were randomized into eight treatment groups as shown in Figure 9. Mean body weight loss was no more than 6% in any treatment group, indicating that the drug treatments were well tolerated (Figure S7). The gedatolisib/fulvestrant/palbociclib triplet combination led to durable tumor regression relative to each single agent and the palbociclib/fulvestrant doublet (Figure 9). Tumors regressed to minimal volumes within 20 days of triplet therapy and did not re-grow, without further therapy, for up to 70 days. At day 20, the triplet combination induced significantly higher tumor growth inhibition (TGI) (132%) than gedatolisib alone (105%, *p* < 0.00001) or the fulvestrant/palbociclib doublet (109%, *p* < 0.00001). The TGI for the doublet combinations of gedatolisib with palbociclib (127%) and gedatolisib with fulvestrant (123%) were also greater relative to each single agent (gedatolisib, 105%; fulvestrant, 83%; palbociclib 88%; *p* < 0.001).

These in vivo studies confirmed the previous in vitro observations and demonstrated that the gedatolisib/fulvestrant/palbociclib triplet induced greater and more durable tumor growth inhibition and regression compared to single- or double-agent treatments.

### 2.9. Effects of the Drug Triplet on BC Cells Adapted to Fulvestrant and/or Palbociclib

Several PI3K, AKT, or mTOR inhibitors combined with ET are approved to treat patients with HR+/HER2- ABC whose disease progressed while receiving prior ET, with or without CDK4/6 inhibitors. To model these patient subpopulations, MCF7 (*PIK3CA* mut) and HCC1428 (*PIK3CA/PTEN* wt) cell lines were cultured with increasing concentrations of fulvestrant (up to 50–100 nM) or palbociclib (up to 1000 nM) to establish cell lines adapted to palbociclib (PalboR) or fulvestrant (FulvR). The PalboR and FulvR cells were further cultured to establish cell lines adapted to both drugs (Palbo/FulvR and Fulv/PalboR, respectively) (Figure S8A).

Based on DNA replication analysis by EdU incorporation assay, the cell lines adapted to palbociclib were approximately 10 times less sensitive to palbociclib, while the cells lines adapted to fulvestrant were at least 100 times less sensitive to fulvestrant relative to the parental lines (see palbociclib and fulvestrant IC_50_ values in Figure S8). The cell lines adapted to both palbociclib and fulvestrant were less sensitive to both drugs, as expected (Figure S8). Further cell viability analyses also showed that the cell lines adapted to palbociclib and/or fulvestrant were more sensitive to gedatolisib growth-inhibitory activity relative to alpelisib, inavolisib, capivasertib, or everolimus (Figure S9).

**Figure 10:**
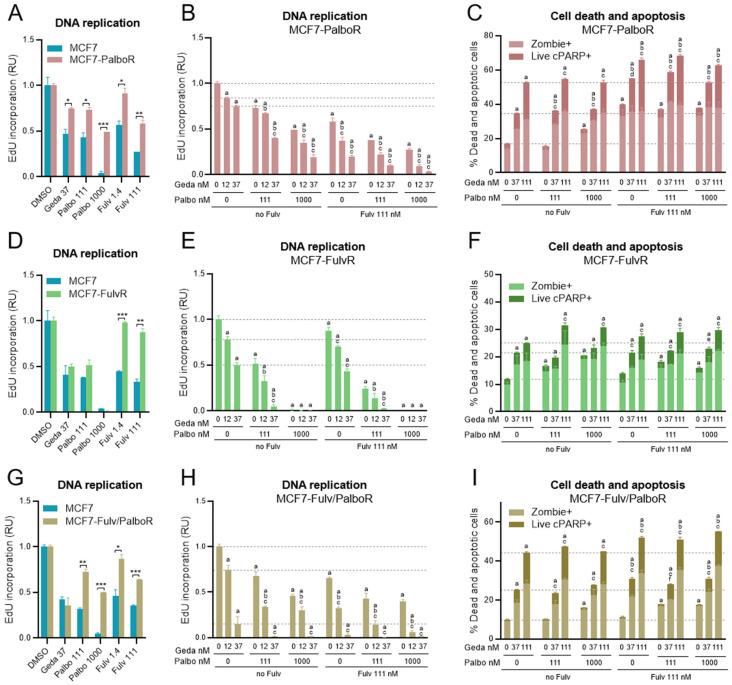
Effects of the gedatolisib/fulvestrant/palbociclib triplet on proliferation and survival of MCF7-derived palbociclib-resistant (PalboR) or fulvestrant-resistant (FulvR) cell lines. (**A**,**B**). Cells were treated with the indicated drugs for 72 h and incubated with EdU for the last 2h of treatment. DNA replication was assessed by flow cytometry analysis of EdU incorporation. The comparison of single drug responses in parental MCF7 and MCF7-PalboR cells is shown in (**A**). The response to various drug combinations in MCF7-PalboR cells is shown in (**B**). Data represent mean +/− standard deviation (*n* = 2). (**C**). MCF7-PalboR cells treated with the indicated drug combination for 72 h were analyzed by flow cytometry for cell death (assessed by Zombie staining) and apoptosis (assessed in live, Zombie-negative cells by staining with anti-cleaved PARP). The graph shows the percentage of both dead cells (Zombie+, % parents) and apoptotic cells (live cleaved PARP+, % grandparents) as mean +/− standard deviation (*n* = 2). (**D**–**I**). Analysis of DNA replication, cell death, and apoptosis in MCF7-FulvR (**D**–**F**) and MCF7-Fulv/PalboR (**G**–**I**) cells as described for (**A**–**C**). For all panels: * *p* < 0.05, ** *p* < 0.01, *** *p* < 0.001 versus MCF7 parental cells; a, *p* < 0.05 vs. DMSO; b, *p* < 0.05 vs. gedatolisib only; c, *p* < 0.05 vs. no gedatolisib within group; d, *p* = 0.05 versus no gedatolisib within group; e, *p* = 0.07 versus no gedatolisib within group; f, *p* = 0.05 versus gedatolisib only by unpaired, two-sided *t*-test. Statistical analysis in (**C**,**F**) and (**I**) refers to the sum of dead plus live apoptotic cells. See Supplementary Data S11 for values.

DNA replication and cell survival in response to 72 h treatment with the gedatolisib/fulvestrant/palbociclib triplet were then evaluated in the adapted cell lines. Similar response trends were observed in the adapted cell lines derived from MCF7 (shown in Figure 10 and Figure S10) or HCC1428 (shown in Figure S11).

Analysis of EdU incorporation demonstrated that, in addition to palbociclib resistance, MCF7-PalboR cells also acquired partial resistance to gedatolisib and fulvestrant (Figure 10A). In these cells, gedatolisib (37 nM), palbociclib (1000 nM), and fulvestrant (111 nM) reduced EdU incorporation by 25%, 51%, and 42%, respectively, as single agents. The gedatolisib/palbociclib, gedatolisib/fulvestrant, and fulvestrant/palbociclib doublets were more effective than the single agents and reduced EdU incorporation by 81%, 80%, and 73%, respectively, while the triplet combination was more effective than each doublet and reduced EdU incorporation by 97% (Figure 10B). The gedatolisib/fulvestrant doublet was also more effective than the single agents at inducing apoptosis and cell death, while the gedatolisib/palbociclib doublet did not significantly increase apoptotic cell death relative to gedatolisib alone (Figure 10C). Interestingly, 111 nM gedatolisib seemed to induce apoptosis and cell death more effectively in MCF7-PalboR (~53%) than parental MCF7 cells (~34%, see Figure 6B). The substantial cytotoxic effects of gedatolisib as a single agent may have partially masked potential combination benefits.

MCF7-FulvR cells maintained similar sensitivity to gedatolisib and palbociclib as parental MCF7 cells (Figure 10D). As such, the highest concentration of palbociclib tested (1000 nM) completely inhibited DNA replication, and the combination effects could only be evaluated with 111 nM palbociclib. The gedatolisib/palbociclib doublet (37 nM/111 nM) reduced EdU incorporation significantly more than the single drugs (95% versus ~50%), while the gedatolisib/fulvestrant doublet (37 nM/111 nM) did not show a significant difference relative to gedatolisib alone (Figure 10E). The fulvestrant/palbociclib doublet (111 nM/111 nM) was more effective than the single agents (75% versus 12–49% inhibition), and the addition of gedatolisib (37 nM) to fulvestrant/palbociclib (111 nM/111 nM) further inhibited EdU incorporation (97%) (Figure 10E). The effect of combining gedatolisib with fulvestrant and/or palbociclib in MCF7-FulvR cells was less significant on cell survival. The gedatolisib/fulvestrant/palbociclib triplet (111 nM/111 nM/111 nM) induced more apoptotic cell death than the fulvestrant/palbociclib doublet (29% versus 18%), but these effects were not significantly different from gedatolisib alone (25%) (Figure 10F).

The MCF7 or HCC1428 cell lines adapted to palbociclib then fulvestrant (Palbo/FulvR), or fulvestrant then palbociclib (Fulv/PalboR), showed similar results to the cell lines adapted to either drug alone (Figure 10G–I, Figure S10 and Figure S11).

These results showed that the gedatolisib/fulvestrant/palbociclib triplet combination was more effective than the single agents or the fulvestrant/palbociclib doublet at blocking DNA replication and/or inducing apoptotic cell death in BC cells adapted to palbociclib and/or fulvestrant, regardless of the presence of *PIK3CA* mutation.

## 3. Discussion

The development of kinase inhibitors for therapeutic intervention has been challenging due to the adaptive nature of kinase signaling pathways in humans. Furthermore, due to the importance of certain kinases, safe therapeutic combinations targeting multiple kinases creates further potential obstacles. The present work describes how a PAM inhibitor targeting multiple PAM pathway kinases could overcome some of the resistance adaptations to current therapies in breast cancer. Treatment with ET in combination with CDK4/6 inhibitors improves the outcome of patients with HR+/HER2- ABC, but most patients eventually develop resistance to the treatment regimens. Our bioinformatics analyses highlighted the concomitant dysregulation of PAM, ER, and CDK pathway activities in the HR+/HER2- BC subtype and the potential of targeting multiple dysfunctions with a PAM pathway inhibitor added to the standard of care with ET and CDK4/6 inhibitors. Since the PAM, ER, and CDK pathways are interdependent drivers of HR+/HER2- advanced breast cancer, the simultaneous inhibition of these three pathways is expected to disrupt their cooperation and counteract resistance. Moreover, as a first-line therapy, the triplet combination of PAM, ER, and CDK4/6 inhibitors could also prevent or delay the onset of resistance upfront, improving outcome in treatment-naïve patients [10]. Here, we tested the triplet combination of gedatolisib, fulvestrant, and palbociclib in BC cell lines to elucidate the mechanisms of their combined activities.

Non-clinical studies have shown that gedatolisib, by targeting multiple PAM pathway kinases and inhibiting key PAM-regulated cellular processes, exerts potent anti-proliferative and cytotoxic effects in cancer cells [36,38,39,40]. A Phase 1b trial reported promising preliminary efficacy of gedatolisib in combination with ET and palbociclib, both in patients who were treatment-naïve for HR+/HER2- ABC and in those previously treated with a CDK4/6 inhibitor [44]. In the present study, we provide non-clinical evidence that the gedatolisib/fulvestrant/palbociclib triplet exerts greater growth-inhibitory effects than any of these drugs when tested as single agents or in various doublet combinations, both in vitro and in vivo. Specifically, the triplet combination reduced the early adaptive response to the single agents and doublet combinations and drove a more effective and durable inhibition of critical cancer cell functions controlled by the PAM, CDK, and ER pathways, such as cell cycle, survival, protein synthesis, and glucose metabolism. In addition, the triplet combination was also effective in BC cells adapted to long-term treatment with fulvestrant and/or palbociclib.

The acquisition of resistance to ET and CDK4/6 inhibitors, like resistance to other cancer drugs, is a multi-step process involving progressive adaptations that can develop into long-term resistance [22,47,48,49]. Resistance to CDK4/6 inhibitors and ET often involves increased activation of kinase signaling pathways, such as the PAM and CDK pathways [12,13]. Our study confirmed that early adaptations to palbociclib in ER + BC cells included re-activation of the PAM pathway as well as restoration of the cyclin D1/CDK-RB pathway and cell cycle progression as previously reported [22]. These early adaptations had longer-term consequences, which were revealed by colony growth after drug removal for about 2 weeks as well as xenograft tumor re-growth after treatment for 21 days. Similar adaptations were also observed in response to fulvestrant, especially in the HCC1428 models, where we observed a 72 h rebound of PAM and CDK-RB-E2F pathway activities and the restoration of cell cycle progression even after co-treatment with the fulvestrant/palbociclib combination. By inhibiting multiple kinases of the PAM pathway, gedatolisib effectively counteracted early adaptations to fulvestrant and/or palbociclib. As a consequence, the addition of gedatolisib to fulvestrant and/or palbociclib reduced cyclin D1/CDK-RB pathway reactivation and cell cycle recovery, resulting in the long-term inhibition of colony expansion in vitro and tumor growth inhibition in vivo.

The concomitant inhibition of the PAM, CDK, and ER pathways by the gedatolisib/ fulvestrant/palbociclib triplet impacted multiple cellular functions critical for tumor progression. Due to the convergence of these three pathways on the cyclin D1/CDK-RB-E2F signaling [10] (see Figure 1), one of the strongest effects of the triplet combination was the compounded inhibition of cell cycle progression. This effect could be due, at least in part, to the ability of the gedatolisib/palbociclib, gedatolisib/fulvestrant, or gedatolisib/fulvestrant/palbociclib combinations to exert a stronger, more durable inhibition of the RB-E2F axis compared to the single agents. In support of this hypothesis, Michaloglou et al. have shown that the combined inhibition of mTORC1/2 and CDK4/6 is necessary to achieve an optimal blockade of the RB-E2F axis and induce long-term inhibition of BC cell growth [25]. In this scenario, the concomitant inhibition of the ER, PAM, and CDK pathways would exert a combined effect on a downstream, bottle-neck function, i.e., the E2F transcriptional function (see Figure 1). However, additional mechanisms not explored in the current study could also be involved; for instance, the inhibition of PI3K-driven MYC expression could reduce CDK2/cyclin E activity downstream of E2F [55].

In addition to blocking cell cycle progression, the triplet combination was also effective in promoting apoptotic cell death. In the MCF7 cell model, the gedatolisib/fulvestrant/palbociclib triplet induced significantly more apoptosis and cell death than gedatolisib alone in vitro. Consistently, the triplet also induced greater and more durable tumor regression than the single agents or each double combination in the MCF7 xenograft model. The inhibition of cancer cell proliferation combined with the induction of cancer cell death could prevent, or at least delay, the development of drug resistance more effectively than other similar combinations that only induce cytostatic effects. We also note that gedatolisib was the agent that contributed the most to induction of apoptosis when combined with fulvestrant and/or palbociclib. In fact, in some of the fulvestrant- and palbociclib-resistant cell lines, the triplet combination only marginally increased cell death compared to gedatolisib alone. In these models, the benefit of adding gedatolisib to palbociclib and/or fulvestrant may be due to the greater inhibition of cell cycle or other cellular functions.

Previous published studies have mostly focused on the effects of PAM, CDK4/6, and ER inhibitor combinations on cell proliferation and, to a lesser extent, cell death [17,22,24,26,27,50]. However, the concomitant targeting of the PAM, CDK4/6, and ER pathways likely affects other cellular functions that could contribute to tumor growth inhibition. Bioinformatics analysis of over 1200 BC patient tumor samples indicated possible associations between the dysregulation of the PAM/CDK/ER pathways in BC progression with upregulated cell cycle-related functions (e.g., E2F-targets, G2/M checkpoint) as well as dysregulation of many other cellular functions, including glycolysis, hypoxia, oxidative phosphorylation, DNA repair, and unfolded protein response. In addition, our previous studies in breast and prostate cancer cell lines have shown that gedatolisib can affect many PAM-controlled cell functions, including protein synthesis and glucose metabolism [39,40]. Other reports have demonstrated even broader effects when PI3K inhibition is especially effective through control of PIP3 production and the effect of reduced PIP3 on the family of GTPases/GEFs [56,57]. Here, we show that the combination of gedatolisib and fulvestrant, with or without palbociclib, inhibited protein synthesis and glucose metabolism significantly more than the single agents or the palbociclib/fulvestrant doublet in breast cancer cells.

The inhibition of protein synthesis by the gedatolisib/fulvestrant doublet or the gedatolisib/fulvestrant/palbociclib triplet could directly affect cancer cell survival and proliferation by depleting cancer cells of essential building blocks necessary to increase their mass and replicate [51,52]. In addition, disrupted translation could lead to decreased levels of specific proteins relevant for the development of drug adaptations and resistance [51,52]. A notable example is cyclin D1, whose upregulation is a relatively common resistance adaptation to both ET and CDK4/6 inhibitors [14,22,28,58,59,60]. Cyclin D1 levels can be modulated through mTORC1-4EBP1-mediated translation [61]. Moreover, PI3K or mTOR inhibitors, by decreasing p4EBP1 and cyclin D1 protein levels, have been shown to restore palbociclib-sensitivity in BC cells [28]. Our data showing that gedatolisib could prevent p4EBP1 recovery and cyclin D1 induction after 72 h treatment with palbociclib suggest that gedatolisib could counteract early adaptations to palbociclib through the inhibition of 4EBP1-mediated cyclin D1 translation.

The inhibition of BC cell glucose uptake and glycolysis by gedatolisib as a single agent or in combination with fulvestrant and palbociclib may yield multiple benefits. First, this approach could directly affect cancer cell survival and proliferation by targeting a well-known vulnerability of cancer cells, i.e., their increased dependence on glucose and glycolysis for energy production and biomolecule synthesis [62,63]. Second, it may enhance efficacy and help overcome drug resistance, for instance, by reducing the acidification of the tumor microenvironment due to lactate production [64,65,66]. In light of these observations, we hypothesize that the increased inhibition of glucose uptake and glycolysis can contribute, at least in part, to the increased growth inhibitory activity of the gedatolisib/fulvestrant doublet or the gedatolisib/fulvestrant/palbociclib triplet. Future studies evaluating the expression or activity of glycolytic enzymes controlled by the PAM and the ER pathways may provide a deeper molecular and physiological understanding of how these drug combinations affect glucose metabolism.

A significant question addressed by our study is whether PAM pathway inhibition (e.g., by gedatolisib) could resensitize cells to CDK4/6 or ER inhibitors after the acquisition of resistance to these drugs. The combination of gedatolisib with palbociclib and/or fulvestrant was more effective than the single agents both in treatment-naïve cells and in fulvestrant/palbociclib-adapted derivatives, suggesting that gedatolisib increased the sensitivity to palbociclib and/or fulvestrant to some degree. The increased sensitivity was more apparent in palbociclib-adapted MCF7 and HCC1428 cells, where the gedatolisib/palbociclib combination induced greater cell cycle inhibition than gedatolisib or palbociclib alone. Moreover, gedatolisib and palbociclib also showed additive growth-inhibitory effects in HCC1806, an RB-positive, *PIK3CA*-amplified, triple negative breast cancer cell line that is relatively resistant to palbociclib [67] (Figure S12). This suggests that gedatolisib could partially resensitize BC cells with intrinsic resistance to palbociclib. Other non-clinical studies have addressed similar questions. Even if there seems to be a consensus that the combination of PAM and CDK4/6 inhibitors may be useful to prevent the onset of resistance to CDK4/6 inhibitors, some studies report that PAM pathway inhibition can re-sensitize cells with acquired resistance to CDK4/6 inhibitors [18,28,50], while others report no benefit in using combinations of PAM and CDK4/6 inhibitors once resistance has been developed [22,26]. The results of our study suggest that combination therapies with gedatolisib and CDK4/6 inhibitors, with or without fulvestrant, could be beneficial both in treatment-naïve BC patients and in patients who progressed after treatment with CDK4/6 inhibitors.

Another relevant finding of the present study is that the combination of gedatolisib with ET and/or CDK4/6 inhibitors was effective in treatment-naïve and palbociclib/fulvestrant-adapted BC cell lines with or without *PIK3CA* mutations. These results are consistent with findings from a Phase 1b clinical trial that showed the gedatolisib/fulvestrant/palbociclib triplet was similarly effective in ABC patients regardless of *PIK3CA* mutations, both in CDK4/6 inhibitor-naïve and CDK4/6 inhibitor-pretreated patients [44]. In addition to canonical *PIK3CA/PTEN* genetic alterations, the increased activation of the PAM pathway in cancer cells can be caused by other genetic and non-genetic factors [1,68,69]. Gedatolisib efficacy across mutant and wild-type *PIK3CA* BC cells could be due to the direct inhibition of multiple kinases of the PAM pathway, including all Class I isoforms of PI3K and the mTORC1/2 complexes. This hypothesis is supported by non-clinical studies showing that gedatolisib was equally effective in breast cancer cell lines regardless of *PIK3CA* mutational status, while alpelisib (targeting only PI3Kα) and capivasertib (targeting only AKT) were less potent and efficacious in breast cancer cell lines with wild-type *PIK3CA* versus mutant *PIK3CA* [39]. A recent study also suggested that targeting mTOR specifically can overcome resistance to ET and CDK4/6 inhibitors in BC cells regardless of their *PIK3CA* mutational status [27].

We previously reported that gedatolisib, by inhibiting multiple PAM pathway kinases and therefore providing better control over potential adaptive mechanisms reactivating the PAM pathway, exerted greater anti-proliferative and cytotoxic effects than inhibitors targeting single PAM pathway kinases (e.g., alpelisib, capivasertib, or everolimus) in breast, prostate, and gynecologic cancer cell lines [38,39,40]. Recent work by an independent group confirmed our findings in breast and endometrial cancer models by combining a dual mTORC1/mTORC2 inhibitor with a PI3Kα inhibitor [70]. In the present study we further showed that gedatolisib combined with fulvestrant and palbociclib exerted greater growth-inhibitory effects than alpelisib, capivasertib, or everolimus combined with fulvestrant and palbociclib in ER + BC cell lines, regardless of the PAM pathway mutational status. These experiments suggest that comprehensive inhibition of the PAM pathway can provide an advantage over the inhibition of single PAM pathway kinases, even in combination with therapies targeting the ER and CDK pathways. We currently do not know the mechanisms providing this advantage; however, based on our previous studies [39] and the results presented here, the combination of fulvestrant/palbociclib with gedatolisib could induce a stronger inhibition of PAM-controlled functions (e.g., cell cycle, survival, protein synthesis, glycolysis) than inhibitors targeting single PAM pathway kinases. In future experiments, it will be relevant to extend these observations to BC cell models adapted to ET and/or CDK4/6 inhibitors.

## 4. Materials and Methods

### 4.1. Bioinformatics Analyses

Clinical, biospecimen, and bulk level RNA-Seq datasets for the TCGA-BRCA dataset (*n* = 60,660 transcripts from 1231 patients, which includes 113 solid tissue normal, 1111 primary solid tumor, and 7 metastatic specimens) were acquired from the Genomic Data Commons (GDC) portal on 1 February 2025. Additional technical information for the samples was integrated from MBatch to facilitate batch-effect correction. The data were filtered to retain only protein-coding transcripts that had matching metadata annotations (*n* = 19,930) and genes with low expression counts (≤10 counts in the smallest sample group) were discarded (the final gene set consisted of 18,251 transcripts). The filtered data were subjected to normalization, variance-stabilizing transformation, and batch-correction using the DESEq2 (version 1.46.0) and limma R packages (version 3.62.2) (input RNA-seq data available upon request). The transformed batch-corrected data were examined for a reduction in the DSC parameter (~0.3 vs. 0.1 after correction) and principal components analysis (PCA), and hierarchical clustering approaches were used to confirm the absence of batch-dependent sample groupings (Figure S1).

We next used row-wise variance-based filtering to select the top 2500 most variable genes in the dataset (matrixStats R package, version 1.5.0), generating input features for downstream clustering models. To identify BC molecular subtypes based on tumor gene expression states, we used the input variable gene list to perform unsupervised k-means clustering (by setting k = 5 based on silhouette analysis, elbow plots of within-cluster sum of squares and gap statistic evaluation and using 25 random starts). Clustering quality was assessed using the factoextra R package (version 1.0.7) for clustering diagnostics. The clustering results were validated through PCA visualization implemented via stats::prcomp(), with sample annotations including PAM50 subtypes [46] and clinical definitions overlaid using ggplot2 to assess the biological relevance of the k-means-based patient clusters.

Following cluster identification, a normal-like cluster was designated as the reference group for subsequent differential expression analysis. Subsequently, we used DESeq2 for conducting pairwise differential expression analyses between each identified cluster and the reference cluster, with default parameters for dispersion estimation and independent filtering. Statistical testing was performed using the Wald test with Benjamini–Hochberg correction for multiple testing. Results were visualized through volcano plots, with differentially expressed genes (DEGs) defined using thresholds of adjusted *p*-value < 0.05 and log2FoldChange > 1.

Biological and functional signatures of transcriptome-based patient clusters were next identified by gene set enrichment analysis (using the fgsea algorithm, with 10,000 permutations and gene set sizes of 15–500), using the msigdbr R package (version 10.0.1) to access curated gene sets from Hallmark (*n* = 50) and KEGG (*n* = 186) repositories. Ranked gene lists generated using DESeq2’s test statistics were assessed for statistical significance using fgsea’s multi-level permutation test (Supplementary Data S1). A customized dot plot to aggregate pathways together with their Normalized Enrichment Scores (NES) and statistics across clusters was created using the ggplot2 R package (version 3.5.1).

Following gene set enrichment analysis of clinical sample clusters, we used a machine learning based method to map cell line samples onto our reduced dimension PCA space. Bulk RNA Seq data in the form of gene level expression counts and associated metadata for 67 breast cancer cell lines were fetched from the DepMap database. We integrated raw counts of cell line samples into the raw counts clinical dataset and reran the data processing pipeline (transformation, batch-correction and PCA) to avoid batch effects that might arise from processing cell line samples separately. At this stage, the Principal Component (PC) coordinates of clinical samples are expected to have shifted slightly due to re-processing with additional samples. To offset this, five elastic net regression models were trained to predict the shift from original coordinates on each of the top 5 PCs. Finally, the models were used to correctly position 9 cell lines of interest onto the original clinical PCA space (Supplementary Data S2). A PCA plot of clinical samples with cell lines overlayed was generated using the ggplot2 R package.

### 4.2. Cell Culture

The BT474, CAMA1, HCC1428, HCC1500, MCF7, MDA175VII, T47D, and ZR751 breast cancer cell lines were obtained from ATCC (Manassas, VA, USA), while EFM19 and HCC1806 were obtained from DSMZ (Braunschweig, Germany). Driver alterations in key PAM pathway genes were identified by analysis of the Cancer Cell Line Encyclopedia (CCLE, Broad 2019 dataset) [71] through cBioPortal (https://www.cbioportal.org/, accessed on 7 July 2022). In the present study, the term ‘wild type’ (wt) is used to define the absence of driver alterations. Cells were authenticated by STR profiling (ATCC) and tested for mycoplasma. Cells were maintained based on the vendor’s recommendations in a 5% CO_2_ humidified incubator at 37 °C. Cells were passaged when sub-confluent and used for experiments within 2–3 passages. The same lot of FBS was used throughout this study, resulting in an E2 concentration of ~2 pmol/L in the growth media used for culture and testing. Palbociclib-adapted (PalboR) and fulvestrant-adapted (FulvR) cell lines were derived from MCF7 and HCC1428 as follows. Each cell line was initially cultured in the presence of the IC_10_ concentration for each drug. The concentration was increased sequentially over several passages until cells were able to expand in the presence of the drug. MCF7 cells were adapted to 1 µM palbociclib or 100 nM fulvestrant; HCC1428 were adapted to 1 µM palbociclib or 50 nM fulvestrant. The palbociclib-adapted and fulvestrant-adapted cell lines were further cultured with both palbociclib and fulvestrant as described above to obtain cell lines adapted to both drugs (PalboR/FulvR and FulvR/PalboR, respectively). Each cell line was expanded under the maximum drug dose and archived for subsequent experiments. Drug-adapted cell lines were authenticated by STR profiling to confirm their origin.

### 4.3. In Vitro Treatments with Inhibitors

Gedatolisib (Celcuity Inc., Minneapolis, MN, USA), palbociclib, fulvestrant, alpelisib, inavolisib, capivasertib, and everolimus (Selleckchem, Houston, TX, USA) were reconstituted in DMSO and stored in aliquots at −80 °C for long term storage, or at −30 °C for short term storage before cell treatments. For cell viability, growth rate inhibition (GR) metrics, flow cytometry, and glucose uptake and lactate production assays, cells were seeded in two or more replicate wells on white 96-well plates coated with a mixture of collagen 1 (Advance Biomatrix, Carlsbad, CA, USA) and fibronectin (Sigma, Burlington, MA, USA) in 180 µL culture medium and left to attach overnight. After attachment, cells were treated with gedatolisib, fulvestrant, and palbociclib alone or in combination for the indicated time by adding 20 µL of 10× drug freshly diluted in medium. The final media volume after treatment was 200 µL. The only exception to this treatment scheme was for experiments in Figure 3A–D, Figure S3 and Figure S12, in which cells received two sequential drug additions of 20 µL of 11× drug for a final volume of 220 µL. As a control, cells were treated with DMSO in the same amount used for drug treatments. Preliminary experiments comparing treatments in standard medium versus medium containing charcoal-stripped FBS showed similar drug response trends.

### 4.4. Cell Viability Assay

Cell viability was assessed by using RealTime-Glo MT Cell Viability assay (Promega, Madison, WI, USA) as previously described [39]. Cell viability analysis was performed following 6 days of drug treatment with single agents or combinations of gedatolisib, fulvestrant, and palbociclib, using DMSO as a vehicle control. RT-Glo enzyme and substrate were diluted 1:600 in warm medium and 40 µL/well of the solution was added to previously treated 96-well plates (experiments in which the final volume was 220 µL received 44 µL/well of enzyme and substrate mix). Plates were incubated for 1–1.5 h in a cell culture incubator at 37 °C and 5% CO_2_, then RTglo MT luminescence (live cells) was measured using an Infinite M1000 microplate reader (Tecan, Männedorf, Switzerland). After background subtraction, relative viability values were obtained by normalizing the relative light units (RLU) to DMSO-treated cells (set as 1). Dose response curves (DRCs) were plotted in PRISM (GraphPad Software version 10.4.1, San Diego, CA, USA), which was used to calculate IC_50_ values.

### 4.5. Proliferation-Normalized Inhibition of Growth Rate (GR) Assays

Normalized GR inhibition was calculated from RTGlo MT measurements before and after a 72 h treatment as described [72]. Normalized GR inhibition is calculated with the formula GR(c,t) = 2k(c,t)/k(0) − 1, where GR(c,t) is the GR value for a drug at concentration “c” at time “t”, k(c,t) is the growth rate of drug-treated cells, and k(0) is the growth rate of untreated control cells. Anti-proliferative effects are indicated by GR values between 0 and 1; cytotoxic effects are indicated by GR values between −1 and 0; and complete cytostasis is indicated by a GR value = 0. GR_50_ (concentration required to obtain a GR value = 0.5) was calculated based on GR value DRCs plotted in Prism (GraphPad Software version 10.4.1).

### 4.6. Drug Synergy Analysis

Drug synergy analysis was performed using the web-based application SynergyFinder (version 3.0, synergyfinder.fimm.fi) [73]. Two- and three-drug synergies were calculated using the Bliss independence model, which provides a synergy score where <−10 indicates antagonism, −10 to 10 indicates additivity, and >10 indicates synergy.

### 4.7. Flow Cytometry

Cells treated for 24 or 72 h with single inhibitors, combinations of inhibitors, or DMSO were harvested from 96-well plates and analyzed by flow cytometry, as previously described [39]. 5-ethynyl-2′-deoxyuridine (EdU), a nucleoside analog that is incorporated into newly synthesized DNA, was used to assess DNA replication. During the last 2 h of drug treatment, cells were incubated with 10 µM EdU (Thermo Fisher, Waltman, MA, USA). At the end of the treatment, both medium (potentially containing floating dead cells) and cells were collected. The medium was transferred to a deep-well 96-well plate, while the cells were washed with PBS (Corning, Corning, NY, USA) and detached by incubation with 0.25% Trypsin (Corning) + 0.5 mM EDTA (VWR, Radnor, PA, USA). Trypsin was blocked with 0.3% Ovomucoid trypsin inhibitor (Worthington, Lakewood, NJ, USA), and cells were transferred to the same deep-well 96-well plate containing the medium collected previously. Plates were centrifuged at 300 g for 7 min at 4 °C, and the cell pellets were washed with PBS and stained for 15 min at room temperature with Zombie NIR viability dye (Biolegend, San Diego, CA, USA). After washing with PBS + 1% BSA, cells were fixed with 1.6% paraformaldehyde for 10 min at room temperature (Electron Microscopy Sciences, Hatfield, PA, USA) and permeabilized with cold ACS grade methanol (Sigma-Aldrich, St. Louis, MO, USA) for 15 min at 4 °C. For cell cycle analysis, following fixation and permeabilization, cells were assayed for EdU incorporation, phospho-RB, phospho-cyclin D1, and FxCycle. EdU incorporation was detected by using the Click-iT EdU Alexa Fluor 647 kit (Thermo Fisher) per the vendor’s instructions. After the Click-iT reaction, cells were washed with PBS + 1% BSA and stained for 30 min at 4 °C with anti-pRB-PE (Ser807/811) (Cell Signaling, Danvers, MA, USA) diluted 1:50 and anti-cyclin D1-AlexaFluor 488 (Thermo Fisher) diluted 1:200. After washing with PBS + 1% BSA and resuspending in FxCycle Violet Stain for DNA content (Thermo Fisher), samples were run on a Novocyte 3005 (Agilent, Santa Clara, CA, USA) flow cytometer. Data were analyzed by using NovoExpress 1.5.6 (Agilent). Cell debris was first excluded from the analysis by forward and side scatter gating. Subsequently, the Zombie staining was used to gate the live cells, which were analyzed for pRB and cyclin D1 levels (median fluorescence intensity after unstained background subtraction) as well as EdU incorporation (% of EdU+ cells). FxCycle and EdU staining were used for cell cycle analysis. Other antibodies used to assess protein targets by flow cytometry in this study were the following: anti-Cleaved Caspase 3-AlexaFluor 647 (1:50, Cell Signaling), anti-Cleaved PARP (Asp 214)-AlexaFluor 647 (1:20, BD Biosciences, San Jose, CA, USA), pRPS6-Brilliant Violet 421 (1:50, Biolegend), and p4EBP1-AlexaFluor 488 (1:25, BD Biosciences). Protein synthesis was assessed by the Click-iT™ Plus O-propargyl-puromycin (OPP) Alexa Fluor™ 647 kit (Thermo Fisher). During the last thirty minutes of treatment, cells were incubated with 5 µM OPP. After OPP incorporation, cells were harvested, stained with Zombie NIR, fixed, and permeabilized as described above. During processing, the OPP reaction mix was prepared as described in the manufacturer’s protocol using the fluorescent dye at a 1:2000 dilution. Fixed and permeabilized cells were incubated with the OPP reaction mix for 30 min. After normalization to DMSO-treated control cells (set at 1), data were analyzed in PRISM to compare relative target levels between drug treatments.

### 4.8. Glucose Uptake and Lactate

Glucose uptake was measured using the Glucose Uptake-Glo kit (Promega). Briefly, cells were seeded in 96-well plates (Corning) coated with collagen I and fibronectin and allowed to attach for 48 hrs. After 48 h, the medium was removed and replaced with fresh growth media and drugs were added. Cells were cultured for 24 h in the presence of drugs, the conditioned medium was removed for lactate measurements, and the cells were processed for glucose uptake. For measuring lactate levels in conditioned media, 10 µL of collected media was added to 500 µL glucose/lactate hemolyzing solution (EKF diagnostics, Cardiff, UK), mixed by vortexing, and processed on the Biosen R-line instrument. Lactate production was assessed by subtracting baseline medium lactate from the lactate level in the conditioned medium. Glucose uptake measurements were performed following the Glucose Uptake-Glo kit protocol. Briefly, cells were washed twice with PBS then 50 µL of 1 mM 2DG solution was added per well and incubated for 10 min. After 10 min, stop buffer and neutralization buffer were added. A total of 100 µL of 2DG6P detection reagent was added and incubated for 1 h at room temperature. Luminescence was measured using an Infinite M1000 microplate reader (Tecan). Glucose uptake and lactate data were normalized to cell number (assessed by BCA analysis).

### 4.9. Colony Formation Assay

MCF7 cells were seeded in 6-well plates at a low-density of 1500 cells/well and allowed to attach for 48 h prior to drug treatment. Cells were treated for either 72 h or 6 days. The 6-day treatments received a media exchange and fresh drug treatment after the initial 72 h. Colonies were allowed to expand for 2–3 weeks. Endpoint cultures were fixed with methanol and stained with 0.5% crystal violet solution (Sigma Aldrich) prepared in 20% methanol for 30 min at RT. Cells were then rinsed, allowed to dry, and imaged. For the quantification of the crystal violet staining, crystal violet was eluted from the stained colonies with a 33% acetic acid solution and absorbance was read at 590 nM using an Infinite M1000 (Tecan) microplate reader.

### 4.10. Quantitative PCR

Cells were seeded on collagen1/fibronectin-coated 12 well plates at 1.5 × 10^5^ cells/well and left to attach for approximately 24 h. After 24 h attachment, cells were treated with gedatolisib, fulvestrant, and palbociclib alone or in combination for 24 or 72 h. After treatment, culture medium was removed, and RNA was extracted with QuickRNA Microprep kit (Zymo, Irvine, CA, USA) per the manufacturer’s instructions. Up to 1 µg RNA was used for cDNA synthesis using the High-Capacity cDNA synthesis kit (Thermo Fisher). cDNA (40 ng/reaction) was used for qRT-PCR with TaqMan Fast Advanced Master Mix (Thermo Fisher) and Taqman probes for E2F1 (Hs00153451_m1), CDC6 (Hs00154374_m1), PUM1 (Hs00472881_m1), and SYMPK (Hs00191361_m1) (Thermo Fisher) on a QuantStudio 3 thermocycler (Thermo Fisher). Relative mRNA expression was calculated based on the ΔΔCt method [74] using both PUM1 and SYMPK as reference genes for normalization.

### 4.11. Animal Studies

All applicable animal care and use regulations, guidelines, and policies were followed under approval by the Institutional Animal Care and Use Committee (IACUC) at Pfizer Inc in accordance with the guidelines described in “Guide for the Care and Use of Laboratory Animals” (NRC, 2011). Female severe combined immunodeficiency (SCID) Hairless Outbred (SHO) 6-week-old mice were purchased from Charles River laboratories (Mattawan, MI, USA). The mice were housed under pathogen-free conditions on a 12:12 light–dark cycle, with water and irradiated rodent diet ad libitum. The general health of the animals was monitored by regular body weight measurements, observations of signs of distress, and food and water consumption. Mice were 8 weeks old at the time of MCF7 tumor cell implantation. The MCF7 xenograft model was chosen because it is an ER + BC model extensively used in non-clinical studies testing the effects of anti-estrogen compounds and other targeted therapy drugs. Since this is an immuno-compromised mouse model, the role of the immune cells on tumor growth could not be assessed. The breast cancer cell line MCF7 was cultured in minimal essential medium (MEM) (Innovative Research of America, Sarasota, FL, USA), supplemented with 10% Fetal Bovine Serum (FBS), 1% Penn/Strep/Glutamine, 1% sodium pyruvate, 1% MEM Non-Essential Amino Acids and 0.01 mg/mL Zinc human recombinant Insulin (all supplements from Thermo Fisher). To generate a subcutaneous mammary fat pad xenograft model, 5 million MCF7 cells per mouse in 0.2 mL volume of RPMI 1640 media (Thermo Fisher) were mixed with an equal amount of Cultrex Basement Membrane extract (Corning) and RPMI 1640 serum free media and injected into the area of the second or third ventral mammary glands. A 90-day estradiol pellet (Thermo Fisher) was implanted into the right dorsal area of the mice two days before cell implantation. When the tumor size reached 230 mm^3^, 18 days post-tumor cell implantation, animals (*n* = 10/group) were randomized based on body weight into 8 treatment groups, including vehicle, 10 mg/kg gedatolisib, 10 mg/kg fulvestrant, 30 mg/kg palbociclib, doublet combinations of 10 mg/kg fulvestrant and 30 mg/kg palbociclib, 10 mg/kg fulvestrant and 10 mg/kg gedatolisib, 30 mg/kg palbociclib and 10 mg/kg gedatolisib and the triplet of 10 mg/kg fulvestrant, and 30 mg/kg palbociclib and 10 mg/kg gedatolisib. The sample size (*n* = 10) was used in previously published studies [36]. Treatment groups were separated by cages and individual mice were identified by tagging. Fulvestrant was formulated in peanut oil and dosed subcutaneously (SC) daily for 3 days and then on days 7, 11, and 14; palbociclib was formulated in 0.5% Methyl cellulose (MC) in water and dosed orally (PO) as a suspension once daily (QD × 21); gedatolisib was formulated in 5% dextrose in distilled water (D5W) and dosed intravenously (IV) once every 4 days (Q4D × 5). There were three separate vehicles formulated in peanut oil, 0.5% MC, and D5W. Each vehicle-treated mouse received all three vehicle formulations dosed SC (QD × 3, then days 7, 11, 14), PO (daily), and IV (Q4D), representing the formulations, route, and schedule of fulvestrant, palbociclib, and gedatolisib, respectively. Tumor volumes and body weights were measured twice and once a week, respectively, by unblinded operators. Tumor volumes were measured manually with a battery-operated caliper and calculated using the formula [(Length × Width × Width)/2)] until day 70. TGI was determined by the following formula: %TGI = [1 − (Vtx − Vt0/Vcx − Vc0)] × 100, where Vc, Vt are the means of control and treated groups. X = day X on study and 0 = initial day of dosing. Tumor regression was determined by the following formula: % Tumor regression = −(Vx − V0)/V0 × 100, where V is the mean of tumor volume, X = day X on study and 0 = initial day of dosing. After dosing stopped at day 21, the tumor growth delay (TGD) assessment was conducted where tumor volume was measured until day 70. All animals were included in the analysis until they were sacrificed, and no data points were excluded from the analysis. Partial regression and complete regression are defined as changes of 30% and 80% from the pretreatment tumor volume of each mouse. No unexpected adverse events were observed. Mice were euthanized at specific treatment endpoints or when tumor burden caused distress, using CO_2_ euthanasia according to IACUC-approved protocols.

### 4.12. Statistical Analyses

Statistical significance was calculated using PRISM (GraphPad) or Excel as indicated in the figure legends. Differences were considered significant when *p* < 0.05. For in vivo studies of MCF7 tumors, statistical analysis was performed using a TGI analyzer. The *p*-values were determined using analysis of covariance (ANACOVA) versus vehicle, versus doublets of fulvestrant and palbociclib, fulvestrant and gedatolisib, palbociclib and gedatolisib, and versus triplet of palbociclib, fulvestrant, and gedatolisib.

## 5. Conclusions

An increased activation of the PAM pathway is a common cancer cell adaptation that can contribute to resistance to BC treatment with ET and CDK4/6 inhibitors. The combination of a PAM inhibitor with ET and/or CDK4/6 inhibitors is a promising therapeutic strategy to prevent, delay, or overcome resistance. This non-clinical study shows that the gedatolisib/fulvestrant/palbociclib triplet exerted greater growth inhibitory effects than the single agents or the palbociclib/fulvestrant doublet by inhibiting multiple cellular functions in BC cells, regardless of their *PIK3CA/PTEN* mutational status. Moreover, the gedatolisib/fulvestrant/palbociclib triplet was effective both in treatment-naïve cell lines and in cell lines adapted to ER and/or CDK4/6 inhibitors. This suggests that this combined therapeutic approach, by counteracting early adaptations (e.g., increased PAM and cyclin D1) that can be hard-wired in long-term resistance, could be effective both as a first-line and second-line therapy. Our non-clinical findings provide a mechanistic rationale for conducting clinical trials evaluating gedatolisib in combination with CDK4/6 inhibitors and ET in HR+/HER2- ABC.

## Data Availability

All data are available in the main text or in the Supplementary Materials. The datasets analyzed during the current study are available from the corresponding author upon reasonable request.

## Supplementary Materials

The following supporting information can be downloaded at https://www.mdpi.com/article/10.3390/ijms26125844/s1.

## Abbreviations

The following abbreviations are used in this manuscript:

4EBP1	eukaryotic translation initiation factor 4E-binding protein 1
ABC	advanced breast cancer
BC	breast cancer
cCasp3	cleaved caspase 3
cPARP	cleaved PARP
CCLE	Cancer Cell Line Encyclopedia
CDK	cyclin-dependent kinase
cPARP	cleaved PARP
EdU	5-ethynyl-2′-deoxyuridine
ER	estrogen receptor
FDA	Food and Drug Administration
Fulv	fulvestrant
FulvR	fulvestrant-adapted cells
Fulv/PalboR	fulvestrant/palbociclib-adapted cells
GDC	genomic data commons
Geda	gedatolisib
GPCRs	G protein-coupled receptors
GR	growth rate
GSEA	gene set enrichment analysis
HER2	human epidermal growth factor receptor 2
HR	hormone receptor
IC50	half-maximal inhibitory concentration
IV	intravenous(ly)
Lum A	luminal A
Lum B	luminal B
mTOR	mechanistic target of rapamycin
mTORC1	mechanistic target of rapamycin complex 1
mTORC2	mechanistic target of rapamycin complex 2
Mut	driver mutation
NES	normalized enrichment scores
NIR	near-infrared
OPP	O-propargyl-puromycin
Palbo	palbociclib
PalboR	palbociclib-adapted cells
Palbo/FulvR	palbociclib/fulvestrant-adapted cells
PAM	PI3K-AKT-mTOR
PAM50	prediction analysis of microarray 50-gene classifier
PARP	poly (ADP-ribose) polymerase
PC	principal component
PCA	principal component analysis
PI3K	phosphatidylinositol 3-kinase
PIP2	phosphatidylinositol (4,5)-bisphosphate
PIP3	phosphatidylinositol (3,4,5)-trisphosphate
PO	oral
PTEN	phosphatase and tensin homolog
Q4D	once every 4 days
QD	once a day
RP	retinoblastoma
RPS6	ribosomal protein R6
RTK	receptor tyrosine kinase
RU	relative units
SC	subcutaneous
SCID	severe-combined-immunodeficient
SD	standard deviation
SEM	standard error of mean
SERD	selective estrogen receptor degrader
SSC	side scatter
TGI	tumor growth inhibition
wt	wild type
