# Supplementary material for: Functional Analysis of the PI3K/AKT/mTOR Pathway Inhibitor, Gedatolisib, Plus Fulvestrant with and Without Palbociclib in Breast Cancer Models
Source: Int J Mol Sci. 2025 Jun 18;26(12):5844. doi: 10.3390/ijms26125844 (PMC12193243; doi:10.3390/ijms26125844)
Supplement: Supplementary file 1 [file ijms-26-05844-s001.zip › Broege_ijms-3683463_Supplementary Figures-rev.pdf]

Supplementary Figures

Functional analysis of the PI3K/AKT/mTOR pathway inhibitor, gedatolisib, plus fulvestrant with and without palbociclib in breast cancer models

Aaron Broege[1#], Stefano Rossetti[1#]*, Adrish Sen[1], Ann DeLaForest[1,§], Laura Davis[1], Megan Seibel[1], Arul Menon[2,3], Sydney Stokke[1], Allison Macaulay[1], Jhomary Molden[1], Lance Laing[1]*

[1]Celcuity, Inc. 16305 36th Ave N, Suite 100, Minneapolis, MN 5544
[2] Department of Molecular and Cell Biology, University of California, Berkeley, CA 94720, USA
[3] College of Computing, Data Science, and Society, University of California, Berkeley, CA 94720, USA
[§] Current address: Labcorp, 3301 Kinsman Blvd, Madison, WI, 53704
[#] Equal contribution

*Correspondence:
Lance Laing
llaing@celcuity.com
Stefano Rossetti
srossetti@celcuity.com

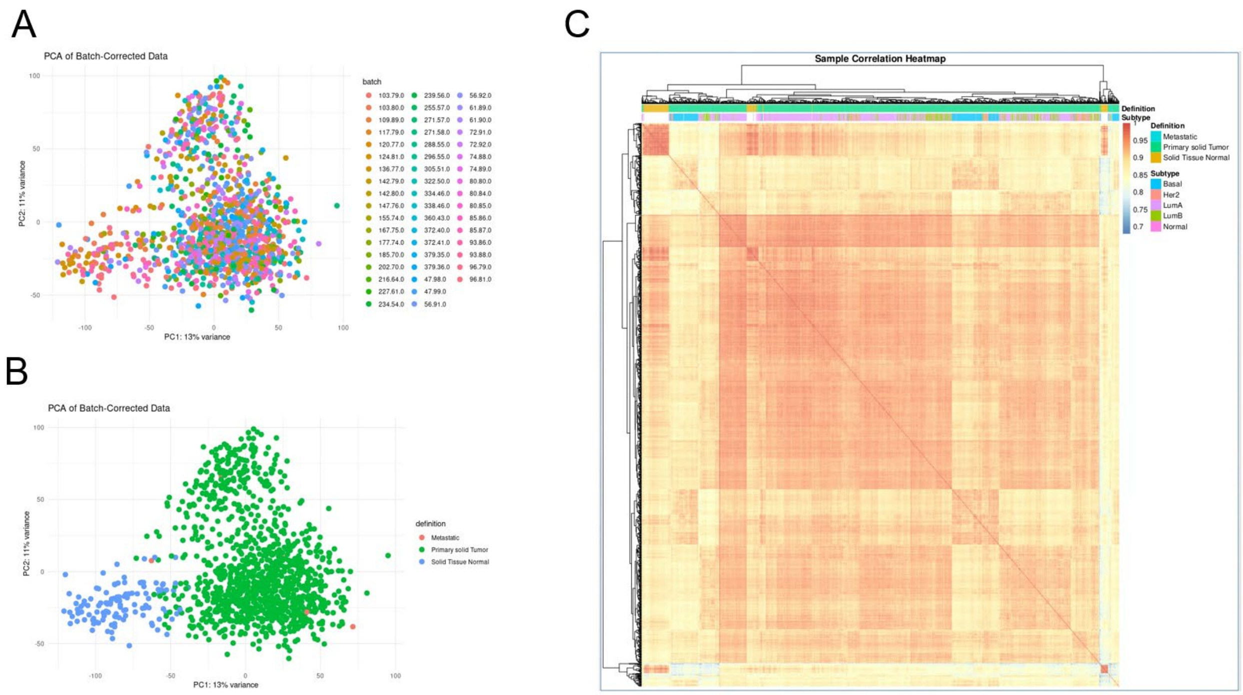

Figure S1. **A-B.** PCA plots of batch-corrected data for expression levels of 18,313 genes from 1,231 BC tumors colored by batch ID (A) or the TCGA definition status (B) demonstrate the lack of batch-dependent sample clustering and retention of biological variability, respectively. **C.** Heatmap of sample distances showing clustering by (the metadata categories) *definition* and *PAM50 subtype*.

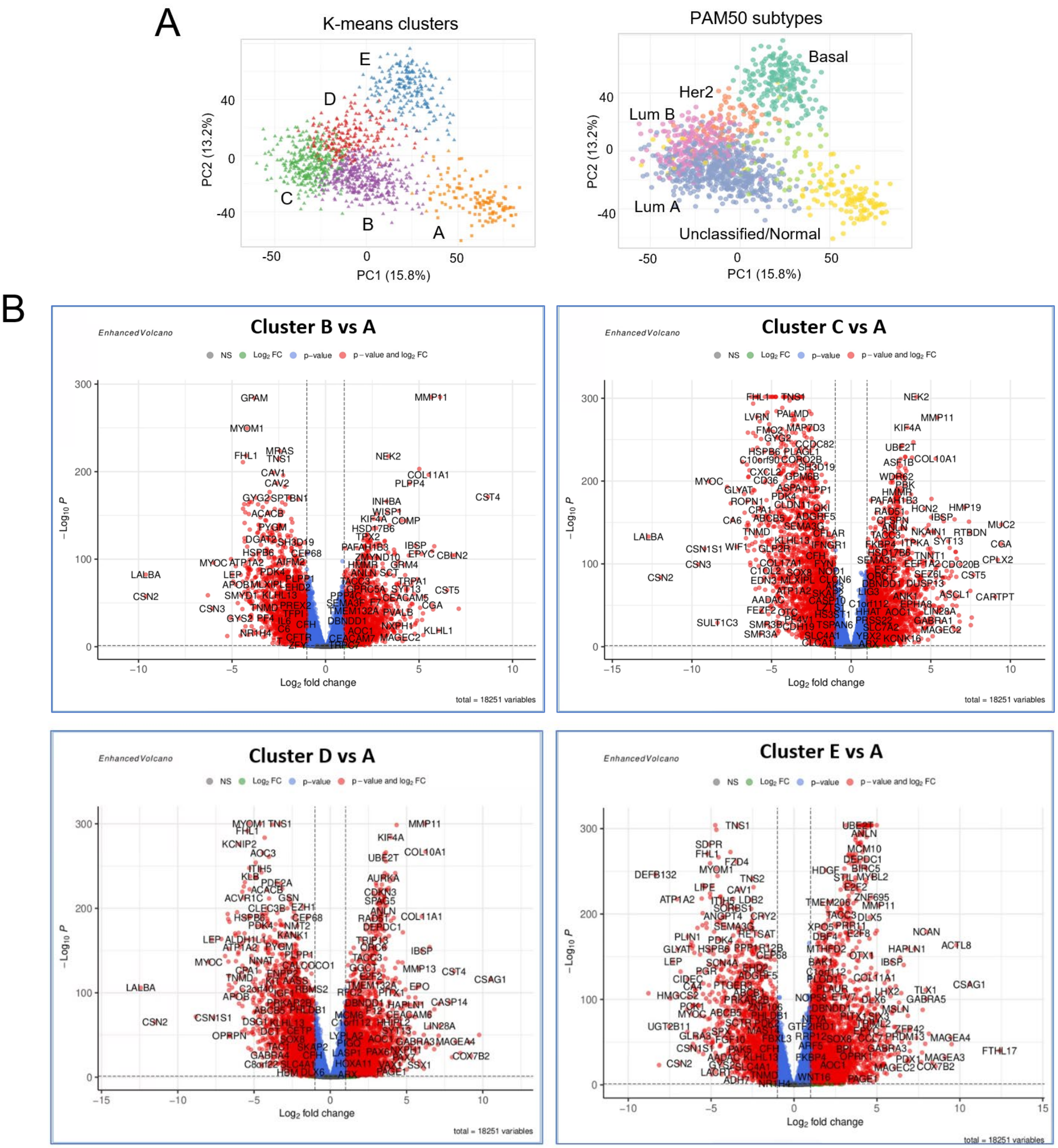

Figure S2. Identification of functional pathways enriched in discrete patient clusters. Individual k-means defined clusters were analyzed for pathway enrichment using GSEA, using both Hallmark and KEGG pathway sets. **A.** K-Means clusters and PM50 subtypes from Figure 2 are shown here as a reference. **B.** volcano plots showing upregulated and downregulated transcripts in each disease cluster relative to cluster A (Normal).

A

Drug	PAM specificity	Cell-free Assay Ki (nM)					
		PI3Kα	PI3Kβ	PI3Kγ	PI3Kδ	mTOR	AKT1/2/3
**Gedatolisib**	PanPI3K/mTOR	0.4	6	8	6	1	-
**Alpelisib**	PI3Kα	5	>1000	250	290	-	-
**Capivasertib**	AKT	-	-	-	-	-	3/8/8
**Everolimus**	mTOR	-	-	-	-	1.6	-

B

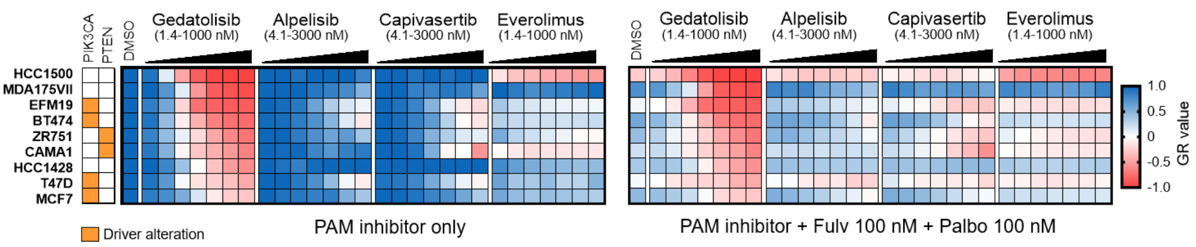

Figure S3. Comparison of gedatolisib alpelisib, capivasertib, or everolimus combined with palbociclib and fulvestrant in BC cell lines. **A.** Specificity and potency of gedatolisib, alpelisib, capivasertib, and everolimus (based on Mallon 2011, DOI: 10.1158/1078-0432.CCR-10-1694; Fritsch 2014, DOI: 10.1158/1535-7163.MCT-13-0865; Addie 2013, DOI: 10.1021/jm301762v; Sedrani 1998, DOI: 10.1016/s0041-1345(98)00587-9). **B.** Growth rate metrics analysis based on cell viability measurement before and after 6-day treatment with increasing concentrations of gedatolisib, alpelisib, capivasertib, and everolimus in the presence of absence of 100 nM fulvestrant and 100 nM palbociclib. GR values > 0 (blue) indicate anti-proliferative effects; GR values < 0 (red) indicate cytotoxic effects. See Supplementary Data S12 for values.

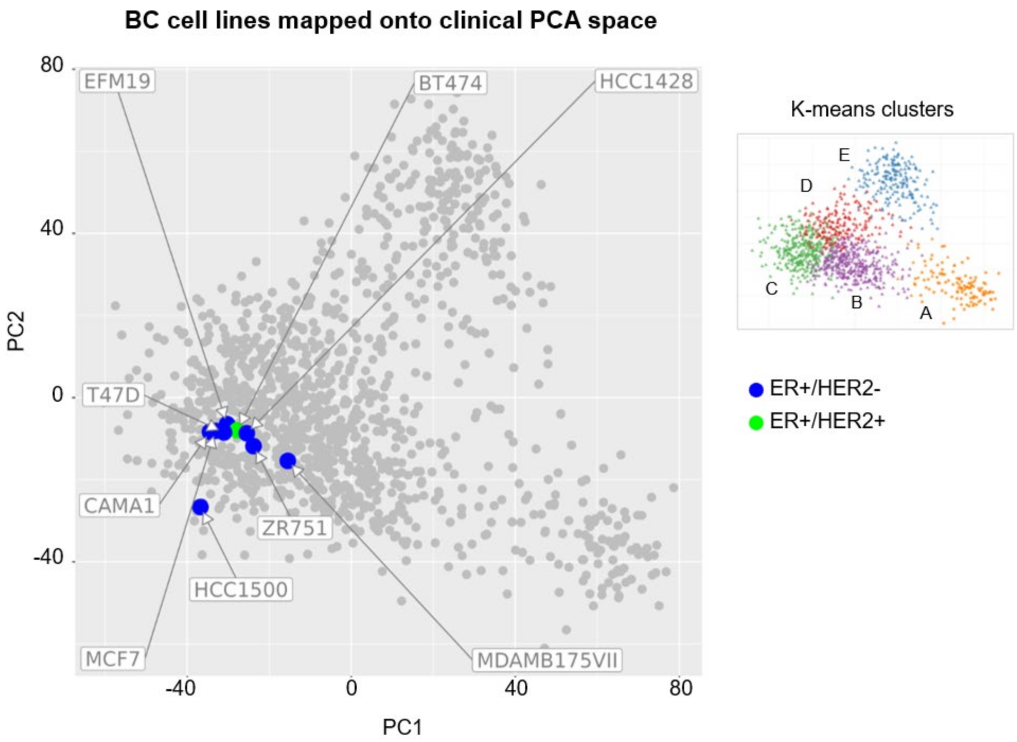

Figure S4. Integration of ER+ BC cell lines into K-means clusters previously identified by transcriptomics analysis of BC samples.

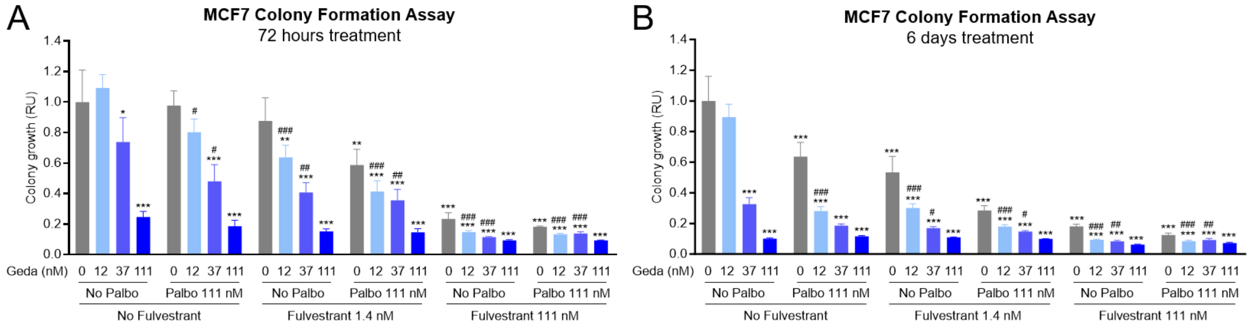

Figure S5. Additional statistical analysis of MCF7 colony formation assay. Cells treated with the indicated concentrations of gedatolisib, palbociclib, and/or fulvestrant for 72 hours (A) or 6 days (B) were allowed to grow for a total of 2-3 weeks until colonies were visible. Colonies were stained with crystal violet (see colony micrographs in Figure 3E and 3F) and eluted to quantify colony growth. *p<0.05, **p<0.01, ***p<0.001 vs DMSO; # p<0.05, ## p<0.01, ### p<0.001 vs gedatolisib by one-way ANOVA. See Supplementary Data S4 for values.

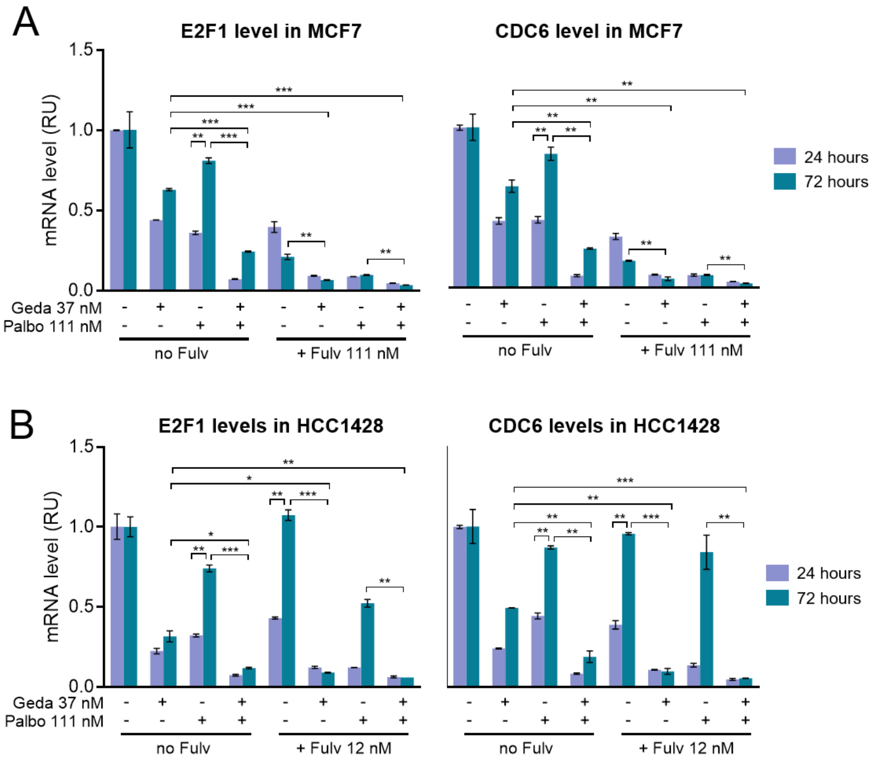

Figure S6. Effects of the gedatolisib/palbociclib/fulvestrant triplet combination on E2F-target genes. **A-B**. qPCR analysis of ER-target genes (E2F1, CDC6) mRNA levels in MCF7 (A) and HCC1428 (B) cells treated with the indicated concentrations of gedatolisib, palbociclib, and/or fulvestrant for 24-72 hours. Data represent mean +/-SD (n=2). * p< 0.05, ** p<0.01, ***p<0.001 by unpaired, two-sided t-test. See Supplementary Data S13 for values.

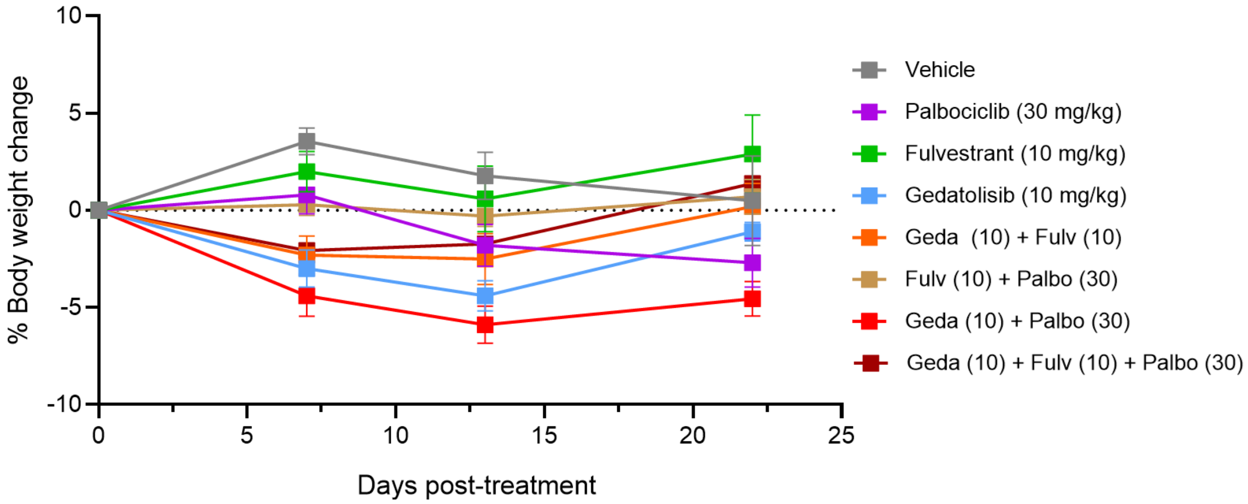

Figure S7. Body weights of SCID mice injected with MCF7 cells in the mammary fat pad and treated with the indicated drugs for 21 days. The chart shows mean body weight change +/- standard deviation for each group.

A

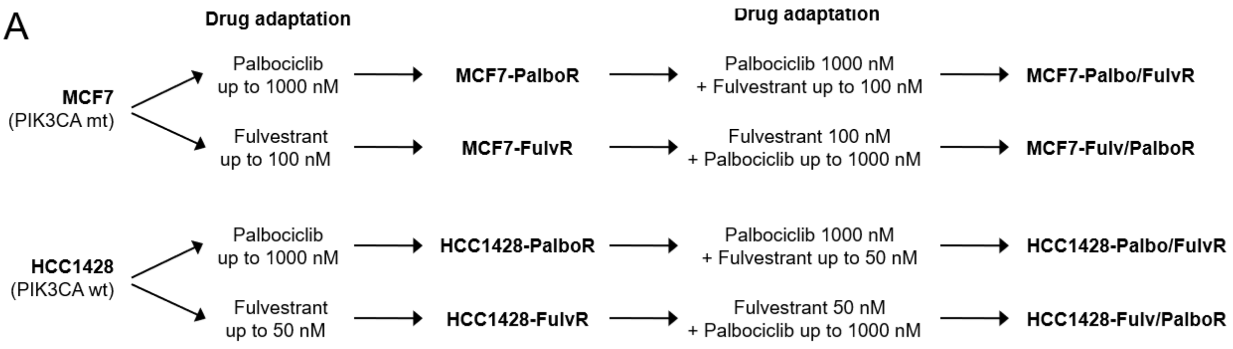

B **MCF7-derived cell lines**

	EdU Abs IC50 (nM)	
	Palbo	Fulv
MCF7	56.3	<1.4
MCF7-PalboR	530.5	72.5
MCF7-FulvR	45.6	>1000
MCF7-Palbo/FulvR	415.4	>1000
MCF7-Fulv/PalboR	861.8	>1000

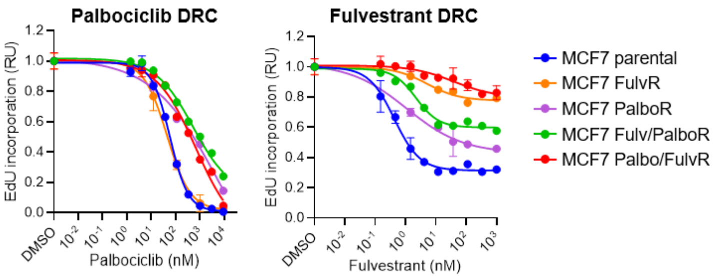

C **HCC1428-derived cell lines**

	EdU Abs IC50 (nM)	
	Palbo	Fulv
HCC1428	222.6	7.3
HCC1428-PalboR	2164	4.0
HCC1428-FulvR	104.5	>1000
HCC1428-Palbo/FulvR	>9000	>1000
HCC1428-Fulv/PalboR	1761	>1000

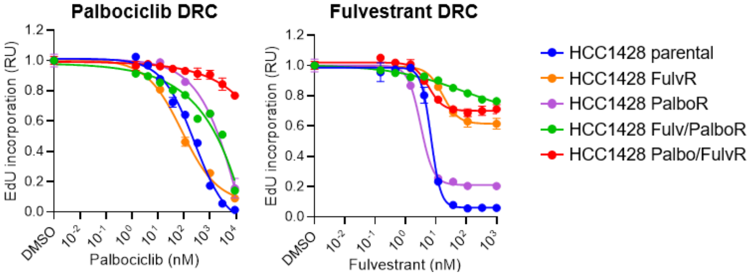

Figure S8. Characterization of palbociclib- and fulvestrant-adapted MCF7 and HCC1428 cell lines. **A.** Scheme summarizing the experimental approach to develop MCF7 and HCC1428 cell lines adapted to palbociclib (PalboR), fulvestrant (FulvR), or both palbociclib and fulvestrant (Palbo/FulvR and Fulv/PalboR. **B-C.** Adapted cell lines derived from MCF7 (B) or HCC1428 (C) were treated with increasing concentrations of palbociclib or fulvestrant for 72 hours and tested for cell proliferation by flow cytometric analysis of EdU incorporation. Dose response curves (shown on the right) were used to calculate absolute IC_{50} values (shown on the left). See Supplementary Data S14 for values.

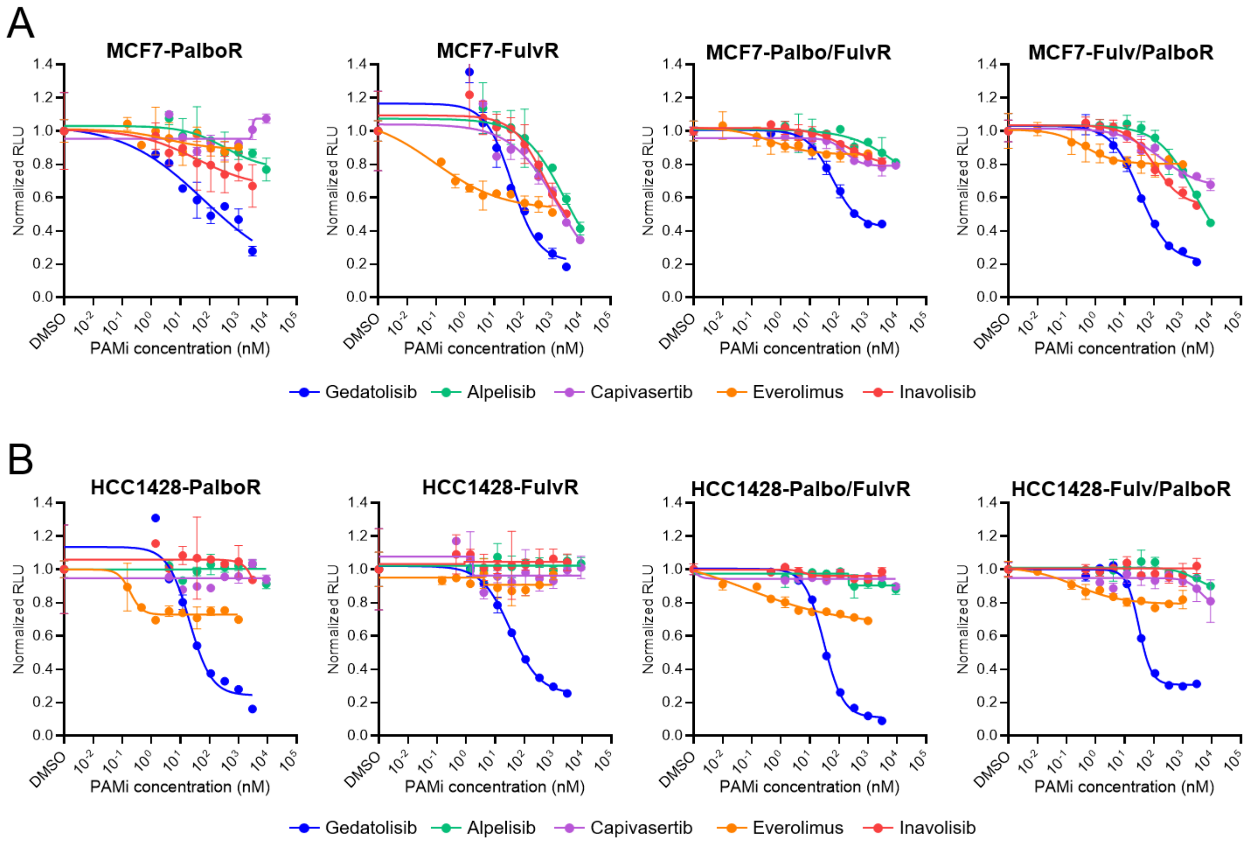

Figure S9. Analysis of cell growth in palbociclib/fulvestrant-adapted MCF7 and HCC1428 cell lines treated with gedatolisib, alpelisib, inavolisib, capivasertib, or everolimus. **A-B.** MCF7 (A) or HCC1428 (B) cell lines adapted to palbociclib and/or fulvestrant were treated with increasing concentrations of gedatolisib, alpelisib, inavolisib, capivasertib, or everolimus for 6 days and analyzed for cell viability by RTGlo MT luciferase assay to obtain dose response curves. Data represent mean +/- standard deviation (n=2). See Supplementary Data S15 for values.

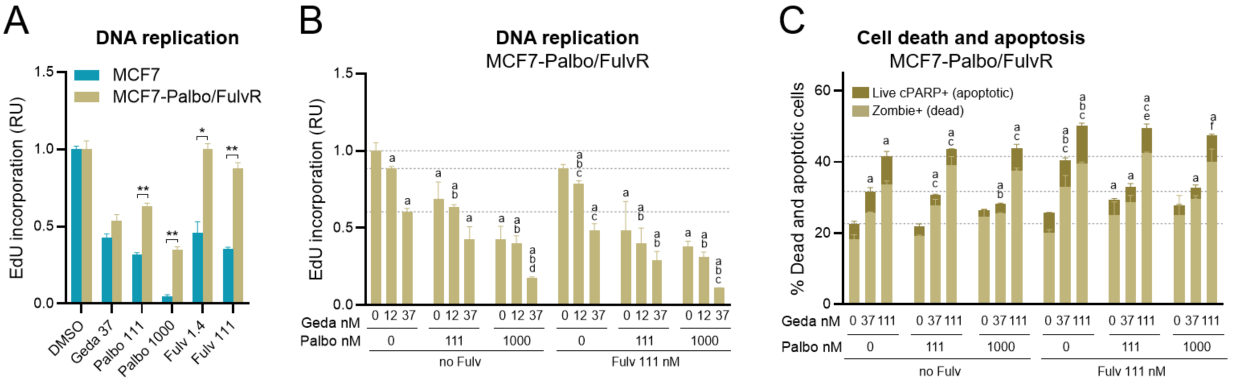

Figure S10. Effects of the gedatolisib/fulvestrant/palbociclib triplet combination on proliferation and survival of MCF7 cells adapted to palbociclib and fulvestrant (Palbo/FulvR). **A-B.** Cells were treated with the indicated drugs for 72 hours and incubated with EdU for the last 2h of treatment. DNA replication was assessed by flow cytometry analysis of EdU incorporation. The comparison of single drug responses in parental MCF7 and MCF7-Palbo/FulvR cells is shown in A. The response to various drug combinations in MCF7-Palbo/FulvR cells is shown in B. Data represent mean +/- standard deviation (n=2-4). **C.** MCF7-Palbo/FulvR cells treated with the indicated drug combination for 72 hours were analyzed by flow cytometry for cell death (assessed by Zombie staining) and apoptosis (assessed in live, Zombie-negative cells by staining with anti-cleaved PARP). The graph shows the percentage of both dead cells (Zombie+, % parents) and apoptotic cells (live cleaved PARP+, % grandparents) as mean +/- standard deviation (n=2). For all panels: * $p < 0.05$, ** $p<0.01$, ***$p<0.001$ versus MCF7 parental cells; a, $p < 0.05$ vs DMSO; b, $p < 0.05$ vs gedatolisib only; c, $p < 0.05$ vs no gedatolisib within group, d, $p=0.050$ vs no geda in group; e, $p = 0.056$ vs gedatolisib only; f, $p = 0.053$ vs no gedatolisib in group by unpaired two-sided t-test. Statistical analysis in C refers to the sum of dead plus live apoptotic cells. See Supplementary Data S11 for values.

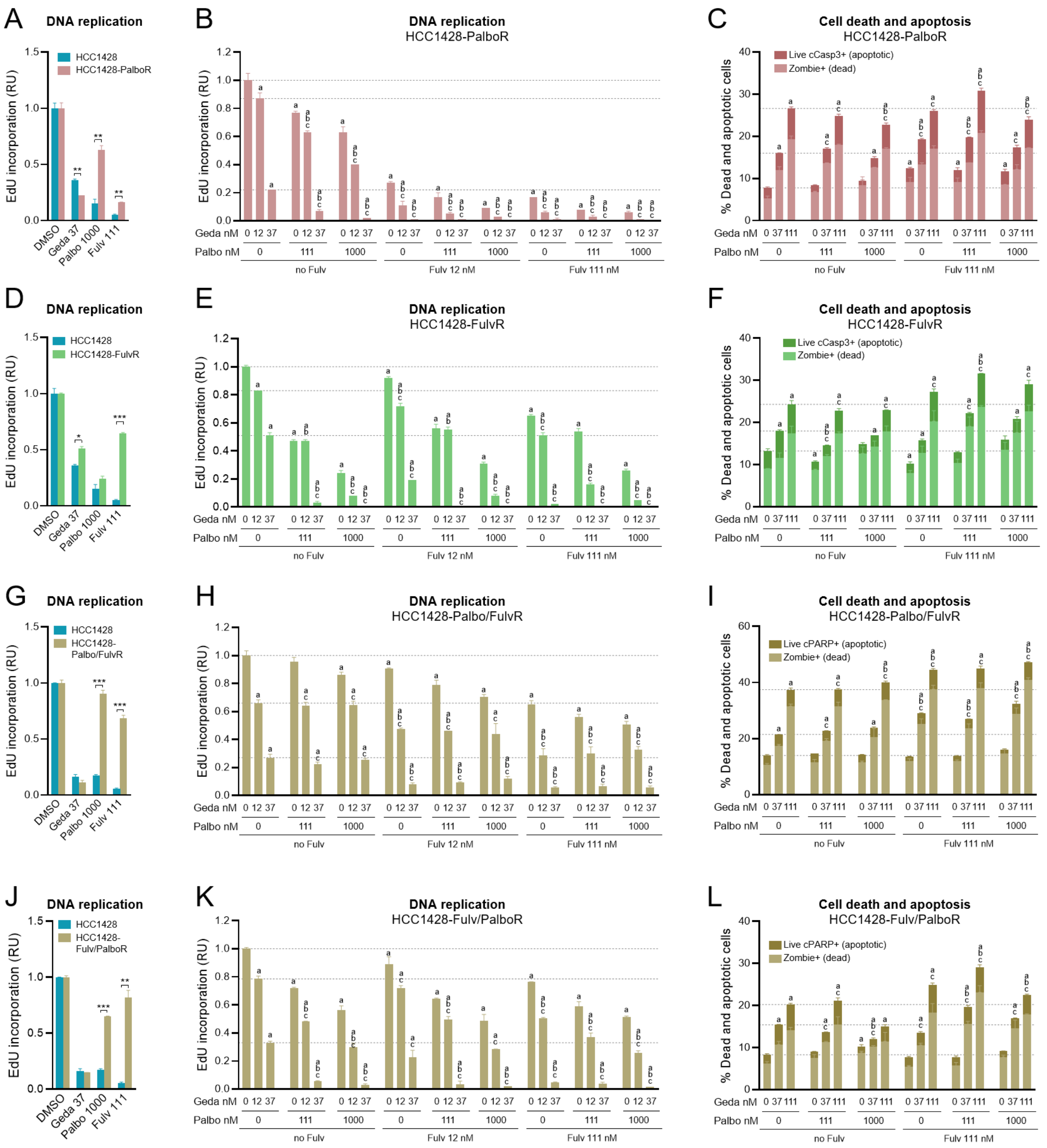

Figure S11. Effects of the gedatolisib/palbociclib/fulvestrant triplet combination on proliferation and survival of HCC1428-derived palbociclib-resistant (PalboR) or fulvestrant-resistant (FulvR) cell lines. **A-B.** Cells were treated with the indicated drugs for 72 hours and incubated with EdU for the last 2h of treatment. DNA replication was assessed by flow cytometry analysis of EdU incorporation. The comparison of single drug responses in parental HCC1428 and HCC1428-PalboR cells is shown in A. The response to various drug combinations in HCC1428-PalboR cells is shown in B. Data represent mean +/- standard deviation (n=2-4). **C.** HCC1428-PalboR cells treated with the indicated drug combination for 72 hours were analyzed by flow cytometry for cell death (assessed by Zombie staining) and apoptosis (assessed in live, Zombie-negative cells by staining with anti-cleaved PARP). The graph shows the

percentage of both dead cells (Zombie+, % parents) and apoptotic cells (live cleaved PARP+, % grandparents) as mean +/- standard deviation (n=2). **D-F.** Analysis of DNA replication, cell death and apoptosis in HCC1428 and HCC1428-FulvR cells as described for A-C. For all panels: * $p < 0.05$, ** $p < 0.01$, *** $p < 0.001$ versus HCC1428 parental cells; a = $p < 0.05$ vs DMSO; b= $p < 0.05$ vs gedatolisib only; c = $p < 0.05$ vs no gedatolisib within group by unpaired, two-sided t-test. Statistical analysis in C and F refers to the sum of dead+live apoptotic cells. See Supplementary Data S16 for values.

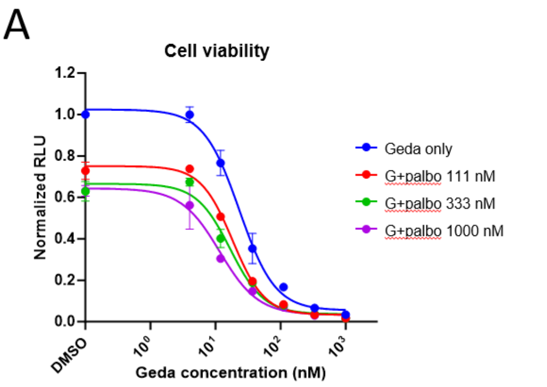

	Geda DRC	Geda DRC + palbo 1000 nM	Palbo DRC	Palbo DRC+ geda 12 nM
Geda IC50 (nM)	26	1.5	NA	NA
Palbo IC50 (nM)	NA	NA	1000-3000	111-333

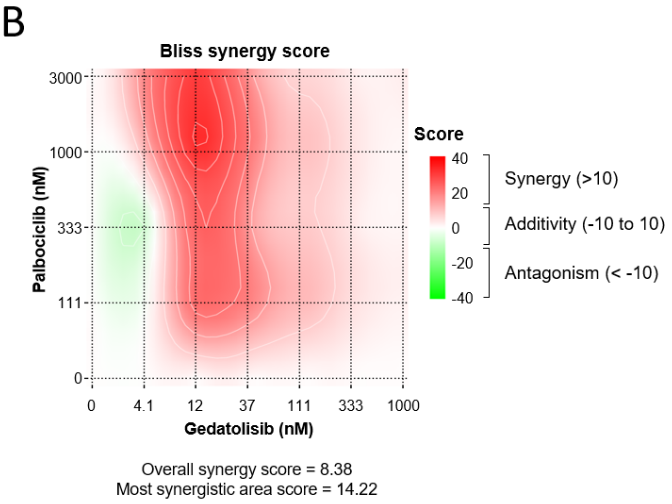

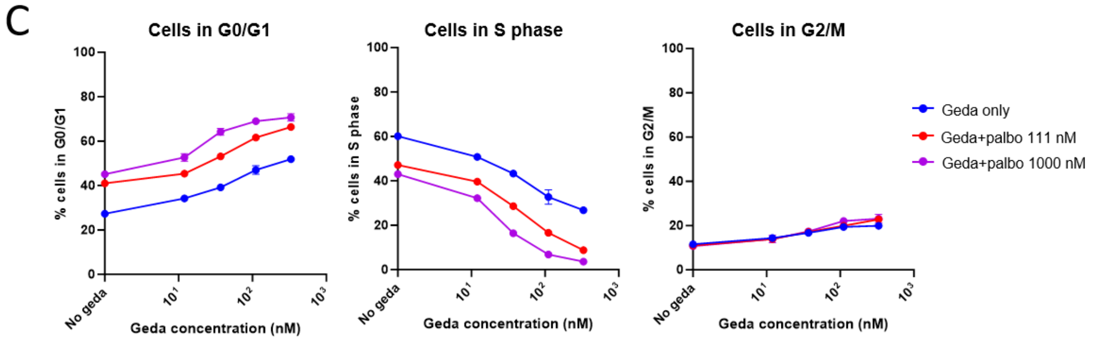

Figure S12. Effects of gedatolisib combined with palbociclib in the HCC1806 BC cell line. **A.** Cells were treated with increasing concentrations of gedatolisib combined with increasing concentrations of palbociclib and analyzed by RTGlo MT cell viability assay to obtain dose-response curves (DRCs, top) and calculate IC$_{50}$ values (bottom). Data represent mean +/- SD (n=2). NA= not applicable. **B.** The cell viability values after treatment with the gedatolisib/palbociclib combinations were used to calculate the Bliss synergy score by SynergyFinder. Scores > 10 indicate synergy; scores between -10 and 10 indicate additivity; scores -10 indicate antagonism. **C.** Flow cytometry analysis of cell cycle phases (identified by DNA staining with FxCycle violet combined with EdU Incorporation assay) in cells treated gedatolisib and palbociclib at the indicated concentrations for 24 hours. Data represent mean +/- SD (n=2). See Supplementary Data S17 for values.
